# Body mass index is associated with pulmonary gas and blood distribution mismatch in COVID-19 acute respiratory failure. A physiological study

**DOI:** 10.3389/fphys.2024.1399407

**Published:** 2024-07-10

**Authors:** Kristín J. Bjarnadóttir, Gaetano Perchiazzi, Caroline Lördal Sidenbladh, Aleksandra Larina, Ewa Wallin, Ing-Marie Larsson, Stephanie Franzén, Anders O. Larsson, Mayson L. A. Sousa, Monica Segelsjö, Tomas Hansen, Robert Frithiof, Michael Hultström, Miklos Lipcsey, Mariangela Pellegrini

**Affiliations:** ^1^ Anaesthesiology and Intensive Care Medicine, Department of Surgical Sciences, Uppsala University, Uppsala, Sweden; ^2^ Hedenstierna Laboratory, Department of Surgical Sciences, Uppsala University, Uppsala, Sweden; ^3^ Anaesthesiology and Intensive Care Medicine, Hudiksvall Hospital, Hudiksvall, Sweden; ^4^ Department of Medical Sciences, Clinical Chemistry, Uppsala University, Uppsala, Sweden; ^5^ Keenan Centre for Biomedical Research, Critical Care Department, St. Michael’s Hospital, Unity Health Toronto, Toronto, ON, Canada; ^6^ Interdepartmental Division of Critical Care Medicine, University of Toronto, Toronto, Canada; ^7^ Translational Medicine Program, Research Institute, Hospital for Sick Children, University of Toronto, Toronto, Canada; ^8^ Section of Radiology, Department of Surgical Sciences, Uppsala University, Uppsala, Sweden; ^9^ Integrative Physiology, Department of Medical Cell Biology, Uppsala University, Uppsala, Sweden

**Keywords:** ventilation/perfusion mismatch, mechanical ventilation, obesity, acute respiratory failure, COVID-19, dual-energy computed tomography

## Abstract

**Background:**

The effects of obesity on pulmonary gas and blood distribution in patients with acute respiratory failure remain unknown. Dual-energy computed tomography (DECT) is a X-ray-based method used to study regional distribution of gas and blood within the lung. We hypothesized that 1) regional gas/blood mismatch can be quantified by DECT; 2) obesity influences the global and regional distribution of pulmonary gas and blood; 3) regardless of ventilation modality (invasive vs. non-invasive ventilation), patients’ body mass index (BMI) has an impact on pulmonary gas/blood mismatch.

**Methods:**

This single-centre prospective observational study enrolled 118 hypoxic COVID-19 patients (92 male) in need of respiratory support and intensive care who underwent DECT. The cohort was divided into three groups according to BMI: 1. BMI<25 kg/m^2^ (non-obese), 2. BMI = 25–40 kg/m^2^ (overweight to obese), and 3. BMI>40 kg/m^2^ (morbidly obese). Gravitational analysis of Hounsfield unit distribution of gas and blood was derived from DECT and used to calculate regional gas/blood mismatch. A sensitivity analysis was performed to investigate the influence of the chosen ventilatory modality and BMI on gas/blood mismatch and adjust for other possible confounders (i.e., age and sex).

**Results:**

1) Regional pulmonary distribution of gas and blood and their mismatch were quantified using DECT imaging. 2) The BMI>40 kg/m^2^ group had less hyperinflation in the non-dependent regions and more lung collapse in the dependent regions compared to the other BMI groups. In morbidly obese patients, gas and blood were more evenly distributed; therefore, the mismatch was lower than in other patients (30% vs. 36%, *p* < 0.05). 3) An increase in BMI of 5 kg/m^2^ was associated with a decrease in mismatch of 3.3% (CI: 3.67% to −2.93%, *p* < 0.05). Neither the ventilatory modality nor age and sex affected the gas/blood mismatch (*p* > 0.05).

**Conclusion:**

1) In a hypoxic COVID-19 population needing intensive care, pulmonary gas/blood mismatch can be quantified at a global and regional level using DECT. 2) Obesity influences the global and regional distribution of gas and blood within the lung, and BMI>40 kg/m^2^ improves pulmonary gas/blood mismatch. 3) This is true regardless of the ventilatory mode and other possible confounders, i.e., age and sex.

**Trial Registration:**

Clinicaltrials.gov, identifier NCT04316884, NCT04474249.

## Introduction

Obesity is a global healthcare problem, and approximately one-fifth of patients admitted to intensive care worldwide are obese (Gong et al., 2010). The obesity paradox describes the observation that, even though obese patients have high overall mortality, they have better intensive care-related outcomes than expected in critical care conditions such as acute respiratory distress syndrome (ARDS) (Ball et al., 2017). One possible explanation is a chronic inflammatory preconditioning characterizing obesity that could induce tolerance for the severe inflammation in critical illness (Stapleton et al., 2010). Another potential explanation is the positive influence of a non-compliant chest wall on the transpulmonary pressure gradient in mechanically ventilated obese patients (Ball et al., 2017).

Most intensive care patients have inefficient pulmonary gas exchange because of poor matching of ventilation and perfusion in the lungs, known as ventilation/perfusion (V/Q) mismatch. Despite its importance, the distribution of V/Q has never been monitored routinely in intensive care, partly due to the methodological complexities (Wagner, 2008). Pulmonary imaging with dual-energy computed tomography (DECT) is a method already widely used in clinical practice to define perfusion defects qualitatively (Godoy et al., 2009; Lu et al., 2012). DECT detects the attenuation profiles of different substances and can potentially quantify the distribution of gas and blood in the lungs at a regional level by using two concurrent X-ray energy spectra. By overlapping the gas and blood distribution maps, regional gas/blood can be estimated as an index of V/Q mismatch.

Our main objectives in this study were: 1) to quantify regional pulmonary gas/blood mismatch using DECT; 2) to assess the influence of obesity on the global and regional distribution of gas and blood in the lungs in patients with acute respiratory failure (ARF); 3) through a sensibility analysis, to study the influence of co-variables other than BMI, i.e., ventilatory modality used (invasive vs. non-invasive ventilation), age and sex (the two latter as indirect indices of comorbidity) on gas/blood mismatch.

Based on the objectives listed above, we hypothesized that 1) the estimation of both global and regional gas/blood match is feasible using DECT; 2) due to different lung mechanics, obesity is associated with less of a pulmonary gas/blood mismatch; 3) regardless of the ventilation modality (invasive vs. non-invasive ventilation) and other potential confounders, patients’ body mass index (BMI) has an impact on pulmonary gas/blood mismatch.

## Materials and methods

This is a single-centre prospective observational study approved by the Swedish National Ethical Review Agency. Written informed consent was obtained from the patients when possible. Otherwise, informed consent was initially asked from next of kin and later confirmed by patients, if feasible. The study was performed in accordance with the ethical standards of the responsible committee on human experimentation and with the Helsinki Declaration of 1975, as most recently amended.

**FIGURE 1:**
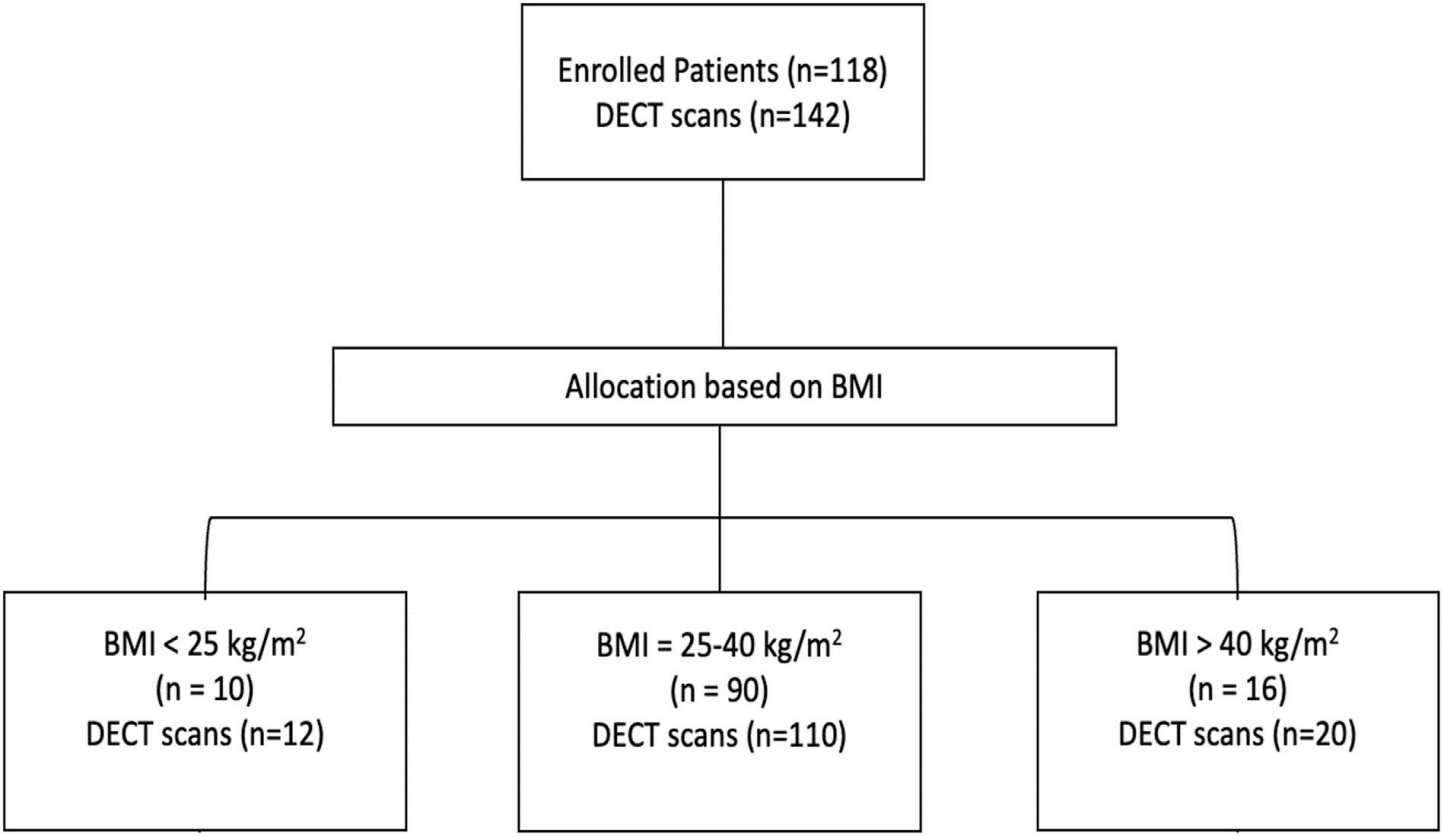
CONSORT flowchart diagram showing the flow of participants through each stage of the study.

The inclusion criteria were 1) age >18 years; 2) a positive polymerase chain reaction test for SARS-CoV2 on a nasal swab specimen; 3) ARF as the main cause of ICU admission, combined with the need for any kind of respiratory support; 4) admission to an ICU at Uppsala University Hospital (Sweden); and 5) at least one chest DECT performed on clinical indication during the ICU stay. Three subgroups were defined according to the patients’ body mass index (BMI) at ICU admission: 1) BMI <25 kg/m^2^ (non-obese), 2) BMI 25–40 kg/m^2^ (overweight to obese), and 3) BMI >40 kg/m^2^ (morbidly obese) (Figure 1). Clinical data were collected at ICU admission, and on the day of DECT.

### DECT data

**FIGURE 2:**
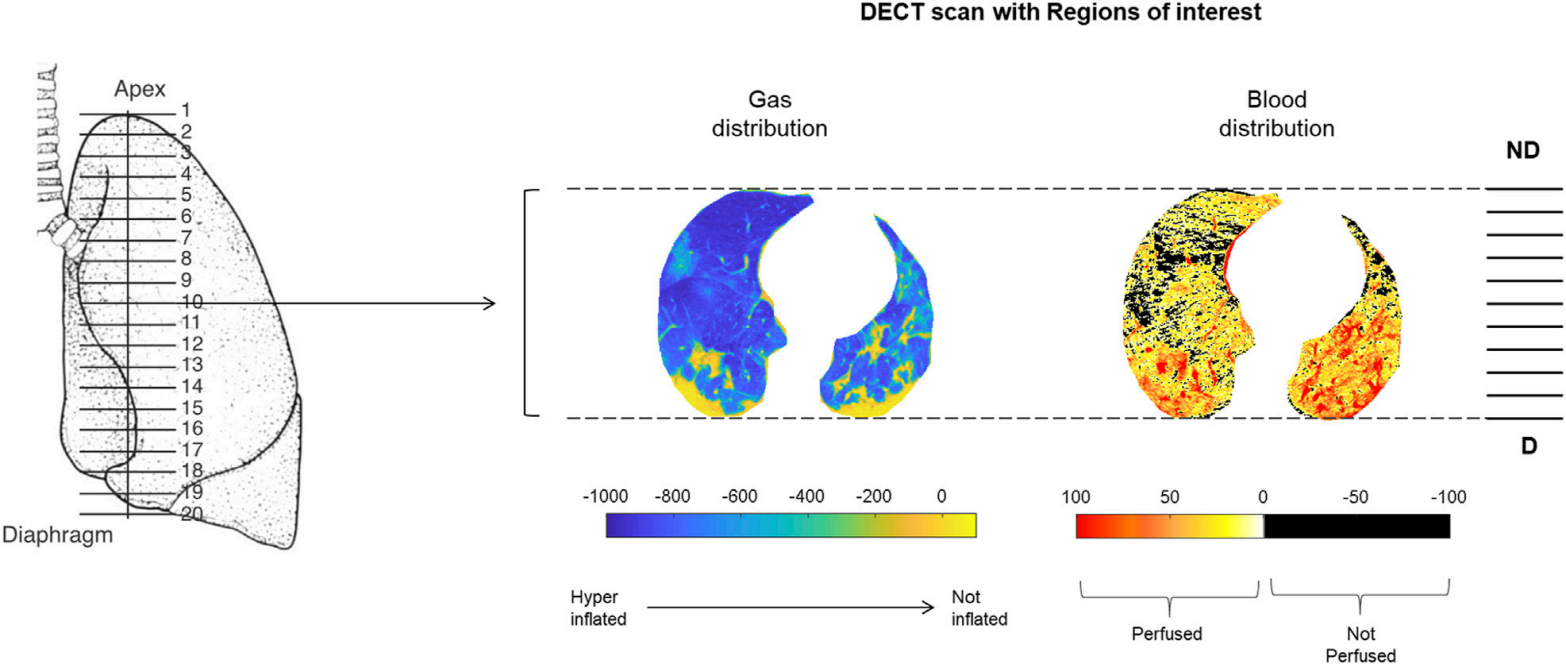
Methods. Representative example of the regions of interest applied to pairs of images. All selected images underwent a semiautomatic delineation of the regions of interest corresponding to the lung parenchyma. The resulting gas and blood distribution maps were subsequently divided into ten gravitational levels. Abbreviations: ND: non-dependent; D: dependent.

DECT scans of patients in a supine position covered the whole lung parenchyma. The DECT software provided paired images reporting gas and blood distribution. From each sequence, 19 equally spaced cranial-caudal images were selected and further divided into ten gravitation levels. To create gas and blood distribution maps, each selected image underwent a delineation of regions of interest corresponding to lung parenchyma. Hounsfield unit (HU) distribution profiles were obtained for the whole lung parenchyma and regionally for each of the ten gravitational levels (Figures 1, 2). Each histogram bin was expressed both as the absolute number of voxels as well as as a percentage of the total number of voxels. Based on the HU distribution profiles, each voxel was respectively defined as hyperinflated, normoinflated, poorly inflated, or noninflated (Gattinoni et al., 2001), and as non-perfused (≤0 HU) or perfused (>0 HU) (Uhrig et al., 2015; Ball et al., 2021; Perchiazzi et al., 2022).

HU peaks for the hyperinflated and noninflated portions were derived from the HU profiles characterising the gas distribution maps. Likewise, the HU peak for blood and the area under the curve were obtained from the HU profiles characterising the blood distribution maps.

The mean HU was calculated for each of the ten gravitational levels and both gas and blood distribution maps. The absolute value of the mean HU is directly correlated to the amount of gas (or blood) in a given lung portion. These absolute mean values were then expressed as a percentage of the sum of the ten HU means composing the whole analysed map. Finally, the difference between the regional percentages of gas and blood was calculated to describe gas and blood mismatch at a regional level.

### Sensitivity analyses

We performed multiple linear regression analyses to investigate the influence on gas/blood mismatch (dependent variable) of: 1) BMI (considered as discrete variable: 5 kg/m^2^), 2) ventilatory modality (invasive vs. non-invasive ventilation), 3) age (years), and 4) sex (male vs. female). To further confirm the effects of BMI on gas/blood mismatch, the same sensitive analysis was also applied to the invasively ventilated and non-invasively ventilated subgroups of patients.

## Results

### Clinical data

Data was collected from 7 April 2020, to 5 October 2021. One hundred eighteen patients were included in the study, and a total of 142 DECTs were performed. In all, 12 (11%) patients were BMI<25kg/m2, where none were under-weight (BMI <18.5kg/m2), 90 (76%) patients were overweight to obese (BMI 25–40kg/m2), and 16 (13%) were classified as morbidly obese with a BMI>40kg/m2 (Figure 1; Table 1). The patients who underwent more than one DECT were evenly distributed among the BMI groups. A majority of the patients were male; however, the BMI>40kg/m2 group had a higher proportion of females than the other two groups. The morbidly obese patients were younger than the other patients included in the study. However, all patients showed a similar incidence of diabetes mellitus and arterial hypertension. Patients with BMI>40kg/m2 were admitted to the ICU after a shorter symptom duration but did not differ from the other groups regarding oxygenation or respiratory rate. The three groups differed regarding ventilatory strategies at the time of DECT (Table 2). Invasive mechanical ventilation was used more often in obese patients, while most of the non-obese patients were either on non-invasive ventilation (50%), on high-flow nasal cannula, or standard oxygen therapy (42%). Among those invasively ventilated, patients with BMI>40kg/m2 were more often on controlled ventilation than those with a lower BMI. Higher minute ventilation characterized patients with BMI <25kg/m2 compared to patients with BMI >40kg/m2. The ratio of arterial oxygen partial pressure to fractional inspired oxygen [PaO2/FIO2] was higher in patients with BMI >40kg/m2, and the alveolar to arterial gradient of oxygen [AaDO2] was lower in the BMI >40kg/m2 group. No differences in pH, carbon dioxide clearance, or cumulative fluid balance were seen among the groups.

**TABLE 1:** Descriptive statistics at admission for the three BMI groups (sample size = 118).

At ICU admission	BMI <25		BMI 25 to 40		BMI >40		Kruskal–wallis/X^2^	1vs2	1vs3	2vs3
Sex [Male/Tot] (n,%)	11/12	92%	72/90	80%	9/16	56%	0.04*	0.30	0.04*	0.04*
Age [years] (median, IQR)	72	15	64	15	56	12	0.01*	0.04*	0.00*	0.15
Arterial hypertension (n,%)	8	66%	54	60%	11	69%	0.72	0.63	0.91	0.48
Diabetes mellitus (n,%)	4	33%	28	31%	6	38%	0.86	0.86	0.82	0.59
	median	IQR	median	IQR	median	IQR				
SAPS-3	56	12	51	11	49	15	0.25	0.68	0.28	1.00
Days of symptoms	10	2	11	3	9	2	0.00*	0.09	1.00	0.00*
Respiratory Rate [bpm]	26	9	25	9	28	9	0.52	1.00	1.00	1.00
PaO_2_/F_I_O_2_ [kPa]	14	10	15	5	16	5	0.90	1.00	1.00	1.00
SaO_2_ [%]	93	5	94	4	94	7	0.98	1.00	1.00	1.00
PCO_2_	4	1	5	1	5	1	0.20	1.00	0.27	0.53
pH	7.47	0.06	7.47	0.05	7.43	0.06	0.35	1.00	0.81	0.48
MAP [mmHg]	86	12	97	21	91	16	0.03*	0.03*	0.38	1.00

Kruskal–Wallis test (α < 0.05) followed by a multiple comparison test with the Bonferroni method for continuous variables (expressed as median and IQR). Alternatively, chi-square (X^2^) test statistic for categorical variables (expressed as number and percent). Abbreviations: BMI: body mass index; SAPS, 3: simplified acute physiology score; PaO_2_: arterial partial pressure of oxygen; F_I_O_2_: fraction of inspiratory oxygen; SaO_2_: arterial oxygen saturation; MAP: mean arterial pressure. Group 1 = BMI<25, Group 2 = BMI, 25 to 40, Group 3 = BMI>40.

**TABLE 2:** Descriptive statistics during DECT for the three BMI groups.

During DECT	BMI <25		BMI 25 to 40		BMI >40		KW/X^2^	1vs2	1vs3	2vs3
Tot number of DECT scans	12		110		20					
Ventilation Strategy							0.15	0.27	0.05*	0.19
Invasive ventilation (n,%)	1	8%	33	30%	10	50%				
Noninvasive ventilation, (n,%)	6	50%	38	35%	6	30%				
High flow nasal cannula or standard oxygen therapy, (n,%)	5	42%	39	35%	4	20%				
Controlled ventilation (n,%)	1	8%	27	25%	7	35%	0.23	0.21	0.09	0.30
	median	IQR	median	IQR	median	IQR				
Days of symptoms before DECT	12	9	14	7	12	9	0.32	0.71	1.00	0.86
Minute Ventilation [L/min]	22	7	13	6	12	5	0.04*	0.02*	0.01*	0.74
PEEP [cmH_2_O]	9	4	12	6	12	5	0.28	0.73	0.34	1.00
Tidal volume/PBW [mL/kg]	12	0	7	3	7	1	0.27	0.35	0.33	1.00
pH	7.45	0.05	7.46	0.08	7.43	0.06	0.14	1.00	0.47	0.58
PaO_2_/F_I_O_2_ [kPa]	14	7	17	8	18	9	0.04*	0.44	0.03*	0.04*
DAaO_2_	43	7	39	14	33	10	0.03*	1.00	0.04*	0.03*
SaO_2_ [%]	93	3	94	4	93	4	0.15	0.86	1.00	0.24
PaCO_2_ [kPa]	4	1	5	2	5	1	0.15	0.46	0.15	0.79
BE	−2	5	2	4	2	3	0.28	0.43	0.42	1.00
Cumulative fluid balance [L]	1	1	1	3	1	1	0.23	0.30	0.34	1.00

Kruskal–Wallis test followed by a multiple comparison test with the Bonferroni method (α < 0.05) for continuous variables (expressed as median and IQR). Alternatively, chi-square (X^2^) test statistic for categorical variables (expressed as number and percent). Abbreviations: PEEP: positive end-expiratory pressure; PBW: predicted body weight; PaO_2_: arterial partial pressure of oxygen; F_I_O_2_: fraction of inspiratory oxygen; DAaO_2_: alveolar–arterial gradient of oxygen; SaO_2_: arterial oxygen saturation; PaCO_2_: arterial partial pressure of carbon dioxide; BE: base excess; BMI: body mass index; SaO_2_: arterial oxygen saturation. Group 1 = BMI<25, Group 2 = BMI, 25 to 40, Group 3 = BMI>40.

### DECT analysis

#### Gas/blood distribution in the whole lung

**FIGURE 3:**
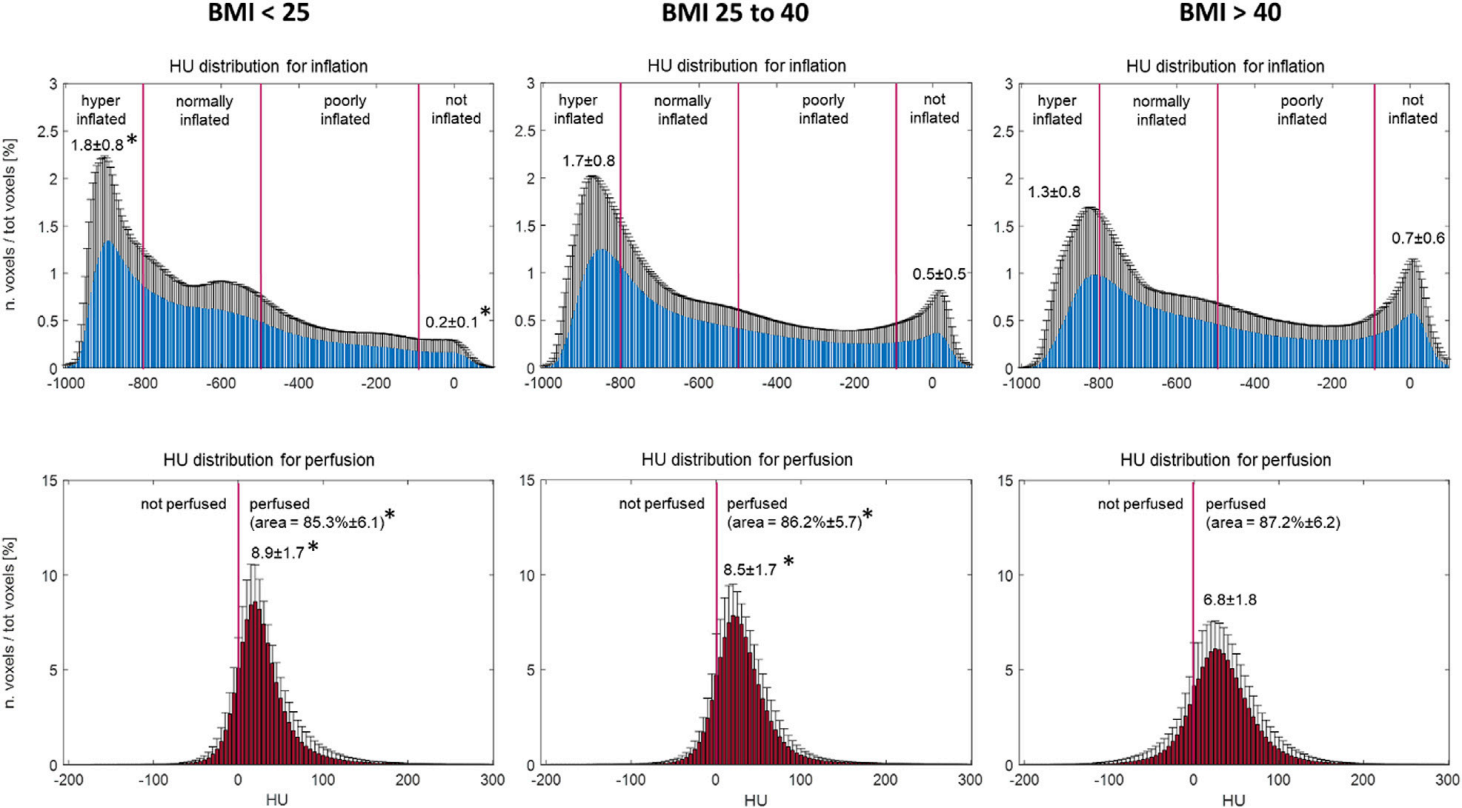
HU distribution for gas (above) and blood (below) maps for the three BMI groups. Histogram bin width equal to 5 HU; for each bin, the values are reported as a percentage of the total voxels (mean ± SD). * To mark differences between the labeled value and the corresponding value for BMI>40. Analysis of variance (ANOVA) followed by multiple comparisons with Bonferroni correction (α < 0.05). Abbreviations: BMI: body mass index; HU: Hounsfield units.

**FIGURE 4:**
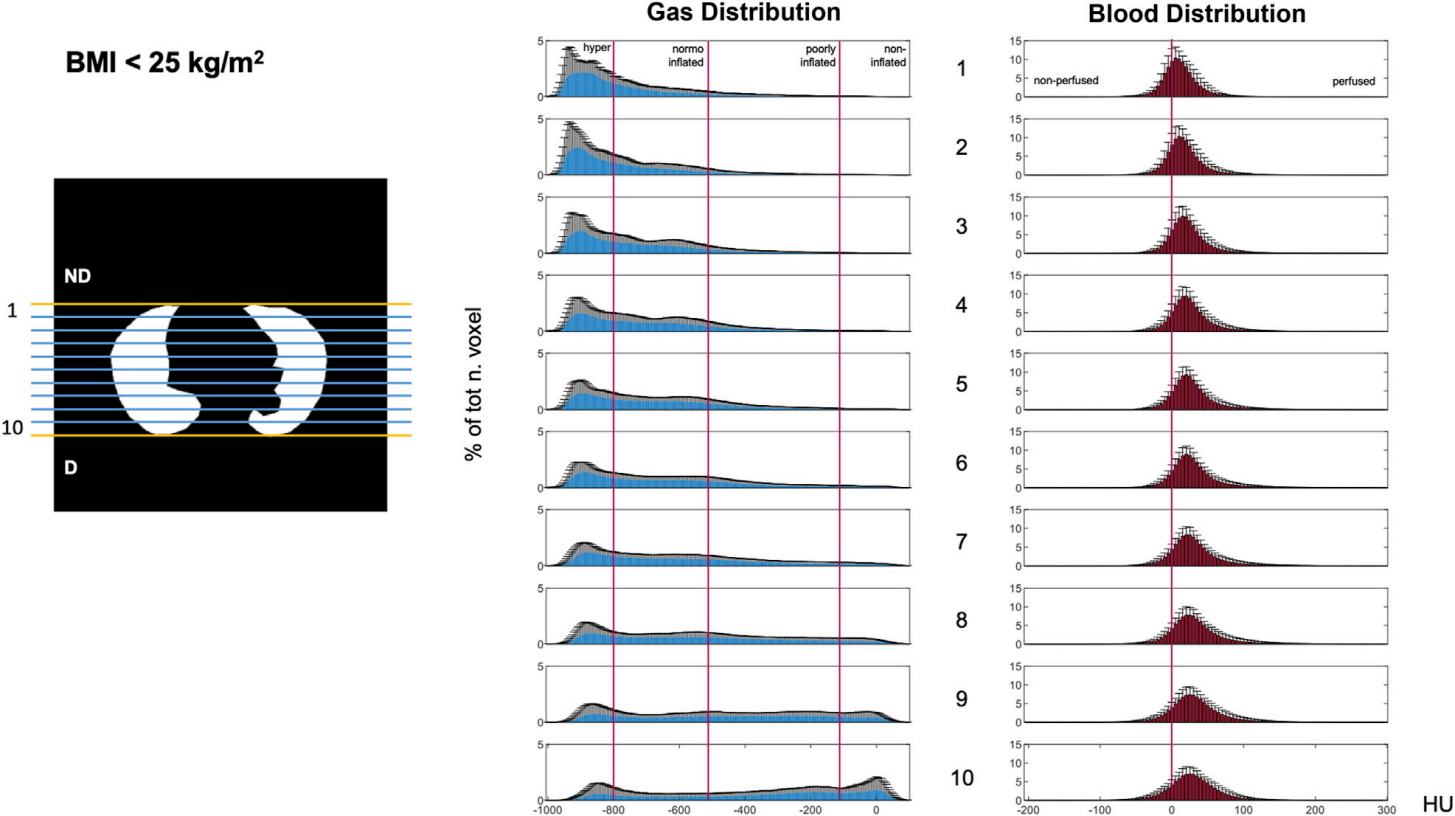
Gravitational HU distribution for gas (left) and blood (right) maps in the BMI<25 group. Data illustrating the ten gravitational levels. Histogram bin width equal to 5 HU; *y*-axis: percentage of total voxels [mean ± SD]. Abbreviations: ND: non-dependent; D: dependent; HU: Hounsfield unit.

**FIGURE 5:**
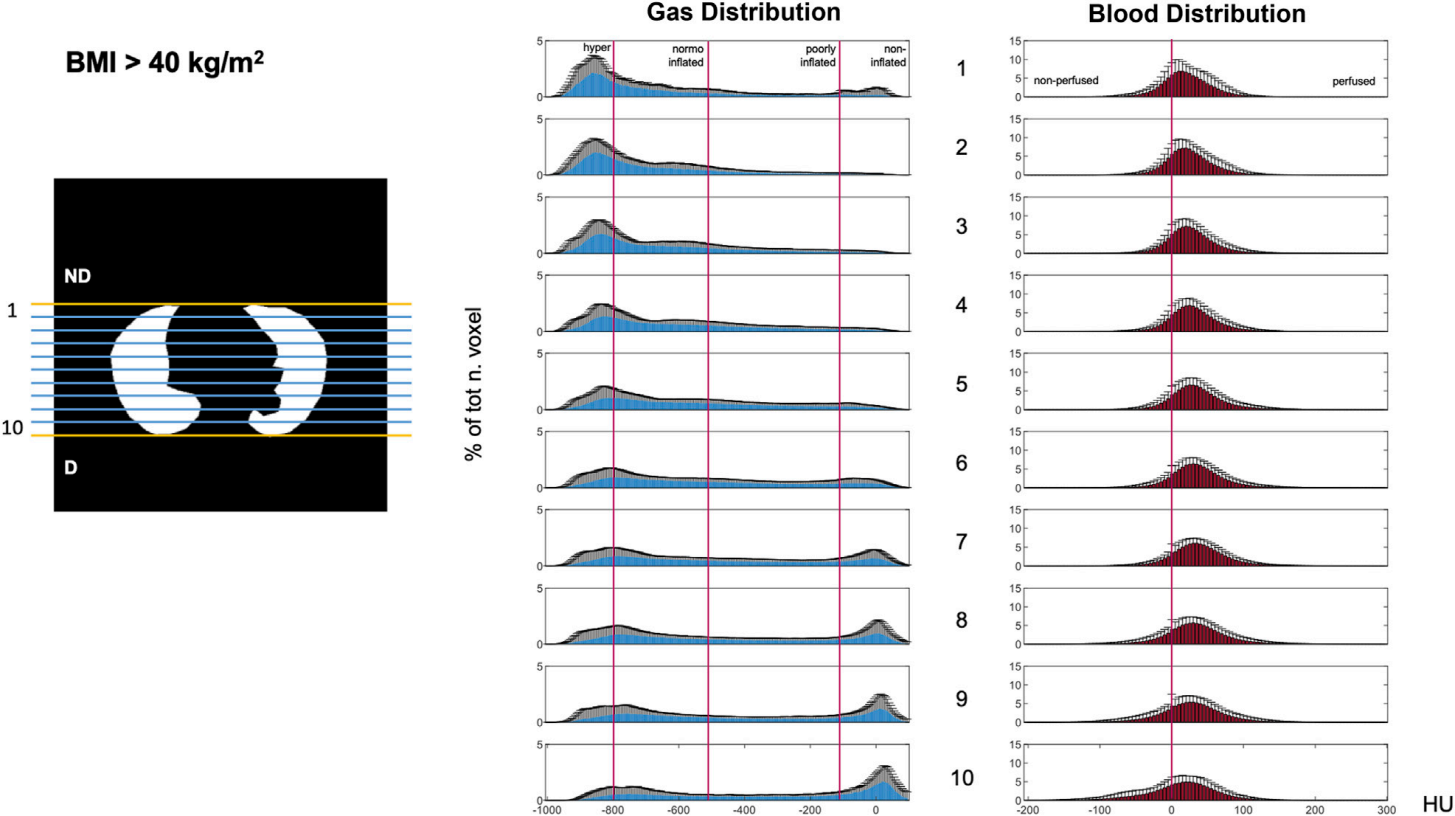
Gravitational HU distribution for gas (left) and blood (right) maps in the BMI>40 group. Data illustrating the ten gravitational levels. Histogram bin width equal to 5 HU; *y*-axis: percentage of total voxels [mean ± SD]. Abbreviations: ND: non-dependent; D: dependent; HU: Hounsfield unit.

The HU distribution profiles derived from the gas distribution maps of the entire studied cohort showed one peak in the hyperinflation HU interval and a second peak in the noninflated HU interval (Figures 2–5). The corresponding blood maps showed a bell-shaped HU distribution. The area under the curve indicating perfused voxels was 85.6% ± 6.3 of the total voxels. The hyperinflated HU was flatter at high BMI. Conversely, the noninflated HU peaked more at higher BMI than at lower BMI. The area under the curve representing the lung’s perfused portion was higher in the BMI >40 kg/m^2^ than in the other BMI groups.

#### Gravitational distribution of gas and blood

**FIGURE 6:**
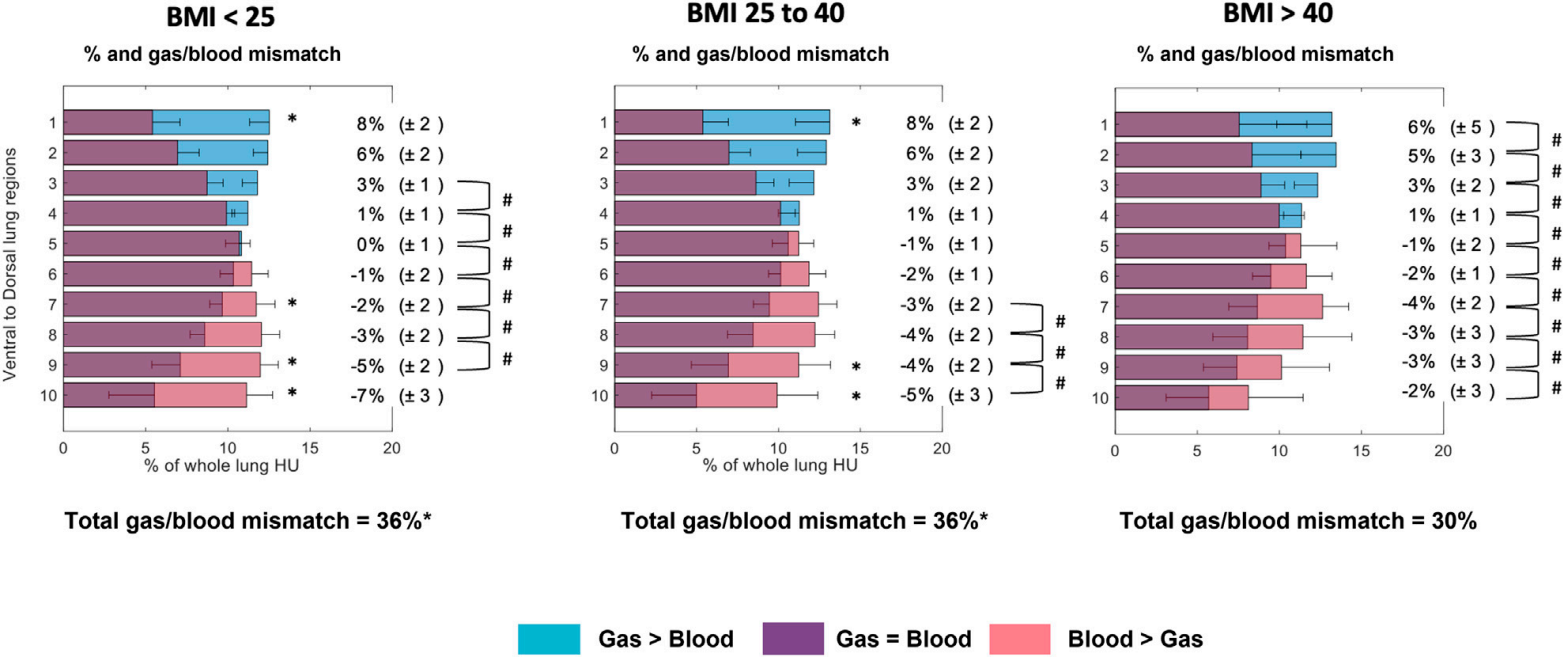
Gas/blood mismatch analysis divided into three BMI groups. Regional 28 distribution is divided into ten gravitational levels for gas/blood match (violet), and 29 mismatch, where blue represents gas exceeding blood and pink represents blood exceeding gas. Compared to the 30 other groups, the BMI>40 group showed a lower, more homogeneously distributed 31 gas/blood match along the gravitational axis. ANOVA followed by multiple comparisons 32 with Bonferroni correction (α < 0.05). * To mark differences between the labelled value 33 and the corresponding value for the BMI>40 group. # To mark differences between 34 contiguous gravitational levels (*p* > 0.05).

Gas and blood were differently distributed along the gravitational axis (Figures 4–6). Gas distribution was most homogenous in the central gravitational levels of the lung (Levels 4–8). The central portion of the lung also showed and a higher amount of blood than the non-dependent and dependent levels, indicating less of a mismatch. A greater part of the lungs was normoinflated in the BMI >40 kg/m^2^ group. Increased BMI was associated with better blood distribution, as indicated by a flat and right-shifted HU distribution.

Total gas/blood mismatch was estimated to be 36% ± 1%. Gas exceeded blood in the most non-dependent lung regions (Figures 5, 6 blue areas, levels 1–4), and blood exceeded gas in the most dependent regions (Figures 5, 6 pink areas, levels 5–10). Patients with BMI>40 showed a lower percentage of gas in the non-dependent regions (Figures 4–6, levels 1–7) than those with lower BMI. Moreover, the BMI>40 group showed higher blood distribution in the most non-dependent regions (Figures 4–6, levels 1–2) and lower blood distribution in the most dependent ones (level 10) than the lower BMI groups.

#### Sensitivity analyses

**FIGURE 7:**
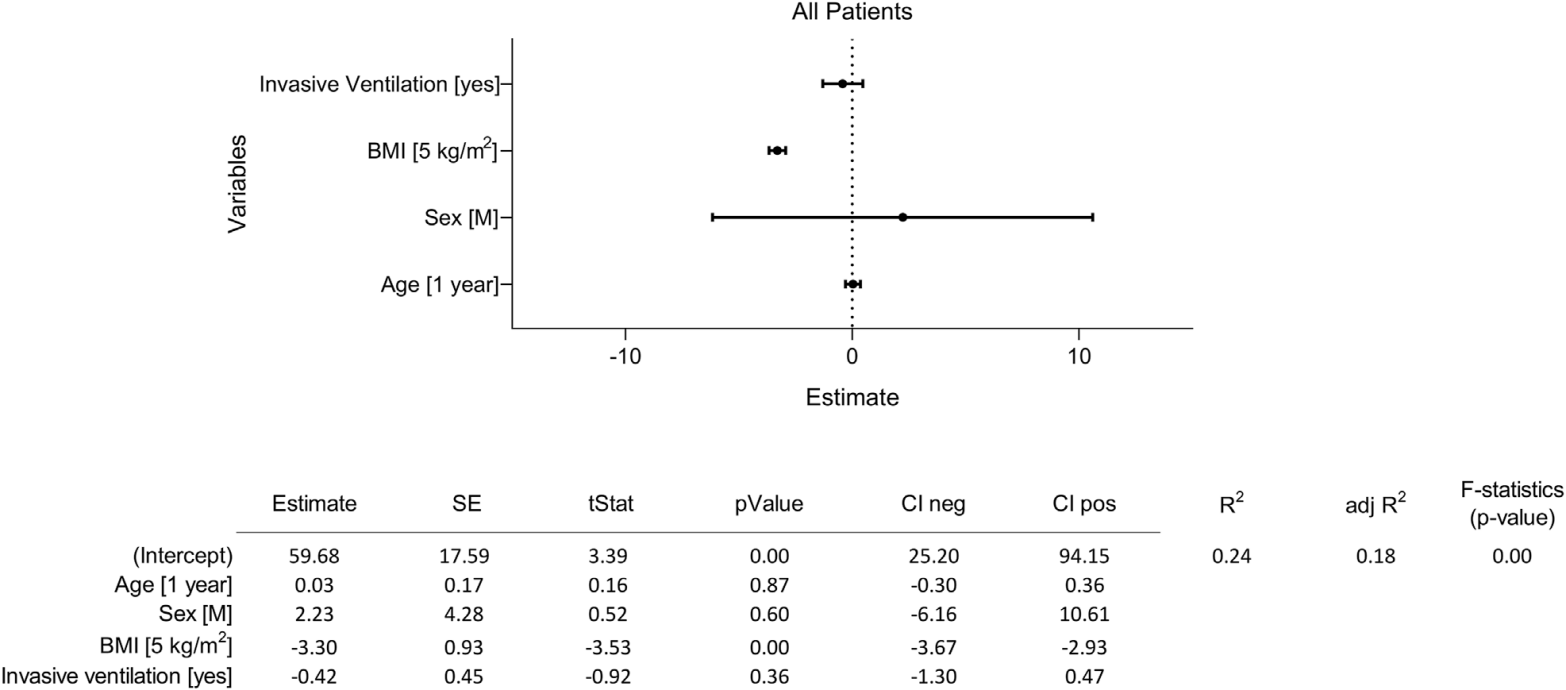
Sensitivity analysis testing the influence of BMI and other potential confounding factors on gas/blood mismatch. The four independent tested variables tested were 1) age (1 year), 2) sex (M), 3) BMI (5 kg/m^2^), and 4) exposure to invasive mechanical ventilation (yes). The multiple linear regression model tested their effect on gas/blood mismatch (dependent variable) based on DECT. Abbreviations: M: male; BMI: body mass index.

**FIGURE 8:**
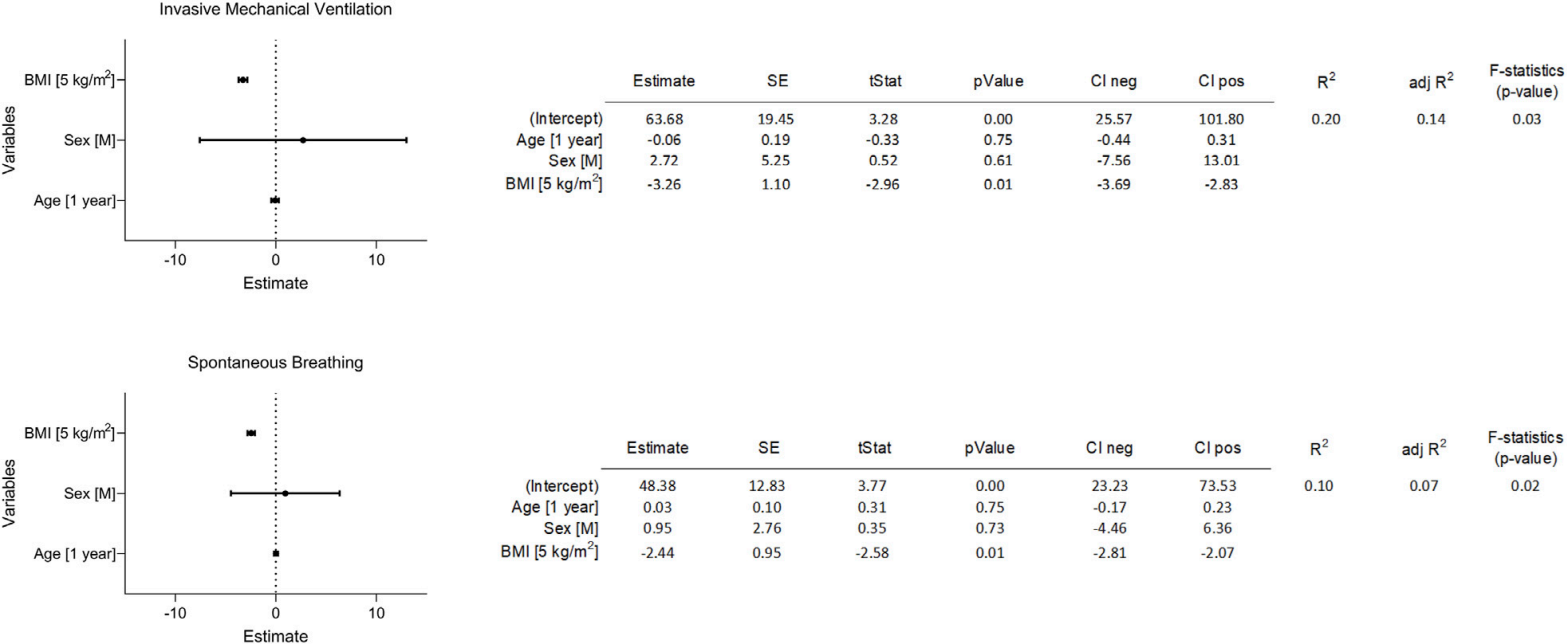
Second sensitivity analysis for two separate subgroups of patients based on the ventilation mode. Sensitivity analysis testing the influence of BMI and other potential confounding factors on gas/blood mismatch based on DECT. The four independent tested variables tested were 1) age (1 year), 2) sex (male), 3) BMI (5 kg/m^2^), and 4) exposure to invasive mechanical ventilation (yes). The multiple linear regression model tested their effect on gas/blood mismatch (dependent variable). In this case the model was applied separately in two subgroups of patients (invasive ventilation vs. spontaneous breathing). Abbreviations: M: male; BMI: body mass index.

A multiple linear regression model was tested to address the association between BMI (as a discrete variable, 5 kg/m^2^) and the gas/blood mismatch, correcting for concomitant variables (i.e., age, sex, and invasive mechanical ventilation). Although BMI resulted in a significant association with the gas/blood mismatch (3.3% reduction in gas/blood mismatch per 5 kg/m^2^ increase in BMI), the multiple linear regression model did not show significant influences of exposure to invasive ventilation, age and sex on gas/blood mismatch (*R* ^2^ = 0.24, *p* < 0.01) (Figure 7). A significant influence of BMI on gas/blood mismatch was confirmed in the invasively and non-invasively ventilated group (respectively 3.3% and 2.4% reduction in gas/blood mismatch per 5 kg/m^2^ increase in BMI, *p* < 0.05 (Figure 8).

## Discussion

The main findings of the current study can be summarized as follows:

1) DECT was used for the first time to quantify, at a global and regional level, gas/blood mismatch in patients affected by hypoxic ARF.
2) Morbidly obese patients affected by COVID-19 ARF and in need of intensive care showed a reduced gas/blood mismatch compared to other patients, a phenomenon not addressed in previous literature (Holley et al., 1967; Hedenstierna and Santesson, 1976; Santesson, 1976; Vaughan and Wise, 1976; Salem et al., 1978).
3) For the first time, this study showed that BMI is associated with an improved gas/blood mismatch in critically ill patients. This was true independently from the ventilatory modality used (invasive vs. non-invasive ventilation) and other possible confounding factors (i.e., age and sex) indirectly representative of patients’ comorbidity.

This study introduced DECT to quantify the pulmonary regional distribution of gas and blood in ARF patients. Despite significant pathophysiological differences between COVID-19 ARDS and other kinds of ARDS (e.g., the physiological response to hypoxemia and the propensity to thromboembolism), our study demonstrates DECT’s untapped potential to provide essential information about pathophysiological causes of impaired gas exchange, which may improve clinical practice.

Despite the extensive literature on the effects of obesity on respiratory mechanics (16), little is known about the impact of obesity on V/Q mismatch (11–15, 17). Moreover, the clinical availability of V/Q monitoring techniques is limited (18), and monitoring regional V/Q distribution in the critical care setting would promote precision medicine. DECT provides static surrogates of V/Q (regional distribution maps of gas and blood) (Gattinoni et al., 2001; Fuld et al., 2013) that may personalize therapeutic strategies, and facilitate more protective mechanical ventilation. Based on DECT analysis, we demonstrated that morbid obesity is associated with improved gas/blood mismatch.

As expected for a cohort of patients affected by ARF (Milic-Emili et al., 1966), the HU distribution showed two typical peaks (Figures 3–5) corresponding to hyperinflated non-dependent and the noninflated dependent (atelectatic) lung compartments (Gattinoni et al., 2001). This regional gradient of gas distribution differed between non-obese and morbidly obese patients (Figures 3–5), with the former showing larger hyperinflation in non-dependent regions and the latter, instead showing more collapsed lung in dependent regions.

The low-BMI group showed less blood in the non-dependent lung regions compared to the high-BMI group. This can be explained by an increase in West Zone I (West et al., 1964), secondary to lung hyperinflation in non-dependent lung zones, leading to an increase in pulmonary vascular resistance, reduced perfusion, and consequently increased alveolar dead space (West, 1978). The low-BMI group generally preserved spontaneous respiratory efforts (Table 2), potentially leading to uncontrolled high tidal volume and high minute ventilation and, therefore, a high risk of self-induced lung injury (Yoshida et al., 2017).

The morbidly obese group mostly showed noninflated dependent areas. This may be a consequence of the increased intraabdominal pressure and loss of functional residual capacity typical of obese patients. Blood was also distributed following a gravitational gradient, except for the most dependent lung regions where content of blood was low, as described by Hughes and West in 1968 (Hughes et al., 1968). Different from gas, blood had a more homogeneous gravitational distribution. Overall, those who were morbidly obese had a more uniform distribution of blood compared to other groups. Interestingly, a similarity could be drawn between the effects of obesity on ventilation distribution and the gas/blood mismatch shown in our study and the therapeutic effects of prone positioning (Gattinoni et al., 2019) and chest wall loading (Selickman and Marini, 2022) in mechanically ventilated patients. In all these three conditions, chest wall displacement is limited, and elastance increased, promoting a lower gravitational gradient of transpulmonary pressure, a more homogeneous distribution of gas, a reduced risk for lung hyperinflation, and improved gas/blood mismatch.

Aware of possible confounders potentially influencing the association between patients’ BMI and changes in gas/blood mismatch, we performed a sensitivity analysis. As possible cofounders, we investigated the ventilatory strategy used (invasive vs. non-invasive ventilation) and patients’ characteristics strictly related to patients’ comorbidities (i.e., sex and age). Moreover, in this case, we looked at the BMI as a discrete rather than as a categorical variable. Although BMI showed a significant influence (a 5 kg/m^2^ increase in BMI was associated with a 3.3% increase in gas/blood mismatch), other covariables did not significantly influence the gas/blood mismatch (Figure 7). These findings were further confirmed by considering the two subgroups of invasively and non-invasively ventilated patients separately (Figure 8).

As previously reported (Anzueto et al., 2011), the morbidly obese patients in our study were younger (Table 1) and had a higher proportion of women. Regardless of comparable comorbidities and clinical presentation, the morbidly obese patients were likely to be admitted to the ICU at an earlier stage of their acute illness. Overall, we found more protective ventilation strategies in patients with high BMI (Table 2). Since the obese population is at high risk for complications, their earlier ICU admission and cautious control of the mechanical ventilation parameters have already been described. (Ball et al., 2017).

## Limitations

The current study has some limitations.

Chest DECT is not a dynamic examination and cannot provide direct information about ventilation. However, CT gas and blood distribution maps are used as a surrogate for ventilation. (Gattinoni et al., 2001; Fuld et al., 2013).

The study cohort included only COVID-19 patients, representing a particular subgroup of ARF patients. Although, at the current state of knowledge, ventilatory management is the same for all ARF patients, the findings of this study need further confirmation in a more general ICU population. However, this does not reduce the physiological relevance of our findings and the potential use of the proposed analysis in the general ICU population. Given the nature of the study, advanced respiratory mechanics data were not collected. Transpulmonary pressure, respiratory system resistance, and chest wall elastance acquired simultaneously with lung imaging could have further confirmed the study’s results.

Body position is essential to optimize ventilation, particularly in obese patients, and a minimum of a 30-degree semi-recumbent position is routinely applied to all patients during intensive care (Wang et al., 2016). The 0-degree supine position during DECT can impact the regional distribution of ventilation. Although this modifies the gas/blood distribution that the patients have in a semi-recumbent position, it is likely that the 30-degree semi-recumbent position improves the gas/blood mismatch to a higher extent in obese patients, further emphasizing the magnitude of our findings.

The inhomogeneous distribution of the human population among the different BMI classes is a characteristic of these specific variables and several other natural phenomena. It is important to highlight that the imbalance among the three selected groups may erroneously be interpreted as a limitation of the study. A standardized stratification of BMI allows an easy interpretation of our results and inference to a clinical context. Moreover, this choice makes our results comparable to previous literature on obese patients.

## Conclusion

Chest DECT can provide information to monitor regional pulmonary gas/blood distribution mismatch in the critical care setting, facilitating personalized therapeutic strategies and more protective mechanical ventilation. Patients’ BMI may affect the gravitational distribution of gas and blood within the lung. Morbid obesity in patients with COVID-19 was associated with reduced gas/blood mismatch which may promote oxygenation, independent of the ventilatory strategy used and other potential confounders such as patients’ age and sex.

## Data Availability

The raw data supporting the conclusion of this article will be made available by the authors, without undue reservation.

## Abbreviations

AaDO_2_, alveolar–arterial gradient of oxygen; ANOVA, Analysis of variance; ARDS, acute respiratory distress syndrome; BE, base excess; BMI, body mass index; D, dependent; DECT, dual-energy computed tomography; FIO_2_, fractional inspired oxygen; HU, Hounsfield unit; ICU, intensive care unit; IQR, interquartile range; ND, non-dependent; PaCO_2_, arterial partial pressure of carbon dioxide; PaO_2_, arterial partial pressure of oxygen; PBW, predicted body weight; PEEP, positive end-expiratory pressure; SAPS-3, Simplified Acute Physiology Score-3; SD, standard deviation; SpO_2_, arterial oxygen saturation; V/Q, Ventilation perfusion ratio; X^2^, chi-square test.

## References

[B1] Anzueto A. Frutos-Vivar F. Esteban A. Bensalami N. Marks D. Raymondos K. (2011). Influence of body mass index on outcome of the mechanically ventilated patients. Thorax 66, 66–73. 10.1136/thx.2010.145086 20980246

[B2] Ball L. Robba C. Herrmann J. Gerard S. E. Xin Y. Mandelli M. (2021). Lung distribution of gas and blood volume in critically ill COVID-19 patients: a quantitative dual-energy computed tomography study. Crit. Care Lond. Engl. 25, 214. 10.1186/s13054-021-03610-9 PMC8215486 34154635

[B3] Ball L. Serpa Neto A. Pelosi P. (2017). Obesity and survival in critically ill patients with acute respiratory distress syndrome: a paradox within the paradox. Crit. Care Lond. Engl. 21, 114. 10.1186/s13054-017-1682-5 PMC5440996 28532465

[B4] Fuld M. K. Halaweish A. F. Haynes S. E. Divekar A. A. Guo J. Hoffman E. A. (2013). Pulmonary perfused blood volume with dual-energy CT as surrogate for pulmonary perfusion assessed with dynamic multidetector CT. Radiology 267, 747–756. 10.1148/radiol.12112789 23192773 PMC3662901

[B5] Gattinoni L. Busana M. Giosa L. Macrì M. M. Quintel M. (2019). Prone positioning in acute respiratory distress syndrome. Semin. Respir. Crit. Care Med. 40, 94–100. 10.1055/s-0039-1685180 31060091

[B6] Gattinoni L. Caironi P. Pelosi P. Goodman L. R. (2001). What has computed tomography taught us about the acute respiratory distress syndrome? Am. J. Respir. Crit. Care Med. 164, 1701–1711. 10.1164/ajrccm.164.9.2103121 11719313

[B7] Godoy M. C. B. Naidich D. P. Marchiori E. Assadourian B. Leidecker C. Schmidt B. (2009). Basic principles and postprocessing techniques of dual-energy CT: illustrated by selected congenital abnormalities of the thorax. J. Thorac. Imaging 24, 152–159. 10.1097/RTI.0b013e31819ca7b2 19465844

[B8] Gong M. N. Bajwa E. K. Thompson B. T. Christiani D. C. (2010). Body mass index is associated with the development of acute respiratory distress syndrome. Thorax 65, 44–50. 10.1136/thx.2009.117572 19770169 PMC3090260

[B9] Hedenstierna G. Santesson J. (1976). Breathing mechanics, dead space and gas exchange in the extremely obese, breathing spontaneously and during anaesthesia with intermittent positive pressure ventilation. Acta Anaesthesiol. Scand. 20, 248–254. 10.1111/j.1399-6576.1976.tb05036.x 785930

[B10] Holley H. S. Milic-Emili J. Becklake M. R. Bates D. V. (1967). Regional distribution of pulmonary ventilation and perfusion in obesity. J. Clin. Invest. 46, 475–481. 10.1172/JCI105549 6021200 PMC442031

[B11] Hughes J. M. Glazier J. B. Maloney J. E. West J. B. (1968). Effect of lung volume on the distribution of pulmonary blood flow in man. Respir. Physiol. 4, 58–72. 10.1016/0034-5687(68)90007-8 5639524

[B12] Lu G. M. Zhao Y. Zhang L. J. Schoepf U. J. (2012). Dual-energy CT of the lung. AJR Am. J. Roentgenol. 199, S40–S53. 10.2214/AJR.12.9112 23097167

[B13] Milic-Emili J. Henderson J. A. Dolovich M. B. Trop D. Kaneko K. (1966). Regional distribution of inspired gas in the lung. J. Appl. Physiol. 21, 749–759. 10.1152/jappl.1966.21.3.749 5912744

[B14] Perchiazzi G. Larina A. Hansen T. Frithiof R. Hultström M. Lipcsey M. (2022). Chest dual-energy CT to assess the effects of steroids on lung function in severe COVID-19 patients. Crit. Care Lond. Engl. 26, 328. 10.1186/s13054-022-04200-z PMC9595078 36284360

[B15] Salem M. R. Dalal F. Y. Zygmunt M. P. Mathrubhutham M. Jacobs H. K. (1978). Does PEEP improve intraoperative arterial oxygenation in grossly obese patients? Anesthesiology 48, 280–281. 10.1097/00000542-197804000-00011 345874

[B16] Santesson J. (1976). Oxygen transport and venous admixture in the extremely obese. Influence of anaesthesia and artificial ventilation with and without positive end-expiratory pressure. Acta Anaesthesiol. Scand. 20, 387–394. 10.1111/j.1399-6576.1976.tb05055.x 793287

[B17] Selickman J. Marini J. J. (2022). Chest wall loading in the ICU: pushes, weights, and positions. Ann. Intensive Care 12, 103. 10.1186/s13613-022-01076-8 36346532 PMC9640797

[B18] Stapleton R. D. Dixon A. E. Parsons P. E. Ware L. B. Suratt B. T. NHLBI Acute Respiratory Distress Syndrome Network (2010). The association between BMI and plasma cytokine levels in patients with acute lung injury. Chest 138, 568–577. 10.1378/chest.10-0014 20435656 PMC2940070

[B19] Uhrig M. Simons D. Ganten M.-K. Hassel J. C. Schlemmer H.-P. (2015). Histogram analysis of iodine maps from dual energy computed tomography for monitoring targeted therapy of melanoma patients. Future Oncol. Lond. Engl. 11, 591–606. 10.2217/fon.14.265 25686115

[B20] Vaughan R. W. Wise L. (1976). Intraoperative arterial oxygenation in obese patients. Ann. Surg. 184, 35–42. 10.1097/00000658-197607000-00006 938116 PMC1344302

[B21] Wagner P. D. (2008). The multiple inert gas elimination technique (MIGET). Intensive Care Med. 34, 994–1001. 10.1007/s00134-008-1108-6 18421437

[B22] Wang L. Li X. Yang Z. Tang X. Yuan Q. Deng L. (2016). Semi-recumbent position versus supine position for the prevention of ventilator-associated pneumonia in adults requiring mechanical ventilation. Cochrane Database Syst. Rev. 2016, CD009946. 10.1002/14651858.CD009946.pub2 26743945 PMC7016937

[B23] West J. B. (1978). Regional differences in the lung. Chest 74, 426–437. 10.1378/chest.74.4.426 699656

[B24] West J. B. Dollery C. T. Naimark A. (1964). Distribution of blood flow in isolated lung; relation to vascular and alveolar pressures. J. Appl. Physiol. 19, 713–724. 10.1152/jappl.1964.19.4.713 14195584

[B25] Yoshida T. Fujino Y. Amato M. B. P. Kavanagh B. P. (2017). Fifty years of research in ARDS. Spontaneous breathing during mechanical ventilation. Risks, mechanisms, and management. Am. J. Respir. Crit. Care Med. 195, 985–992. 10.1164/rccm.201604-0748CP 27786562

