# Supplementary material for: Body mass index is associated with pulmonary gas and blood distribution mismatch in COVID-19 acute respiratory failure. A physiological study
Source: Front Physiol. 2024 Jul 10;15:1399407. doi: 10.3389/fphys.2024.1399407 (PMC11266150; doi:10.3389/fphys.2024.1399407)
Supplement: Supplementary file 1 [file Presentation1.PPTX]

## Slide 1
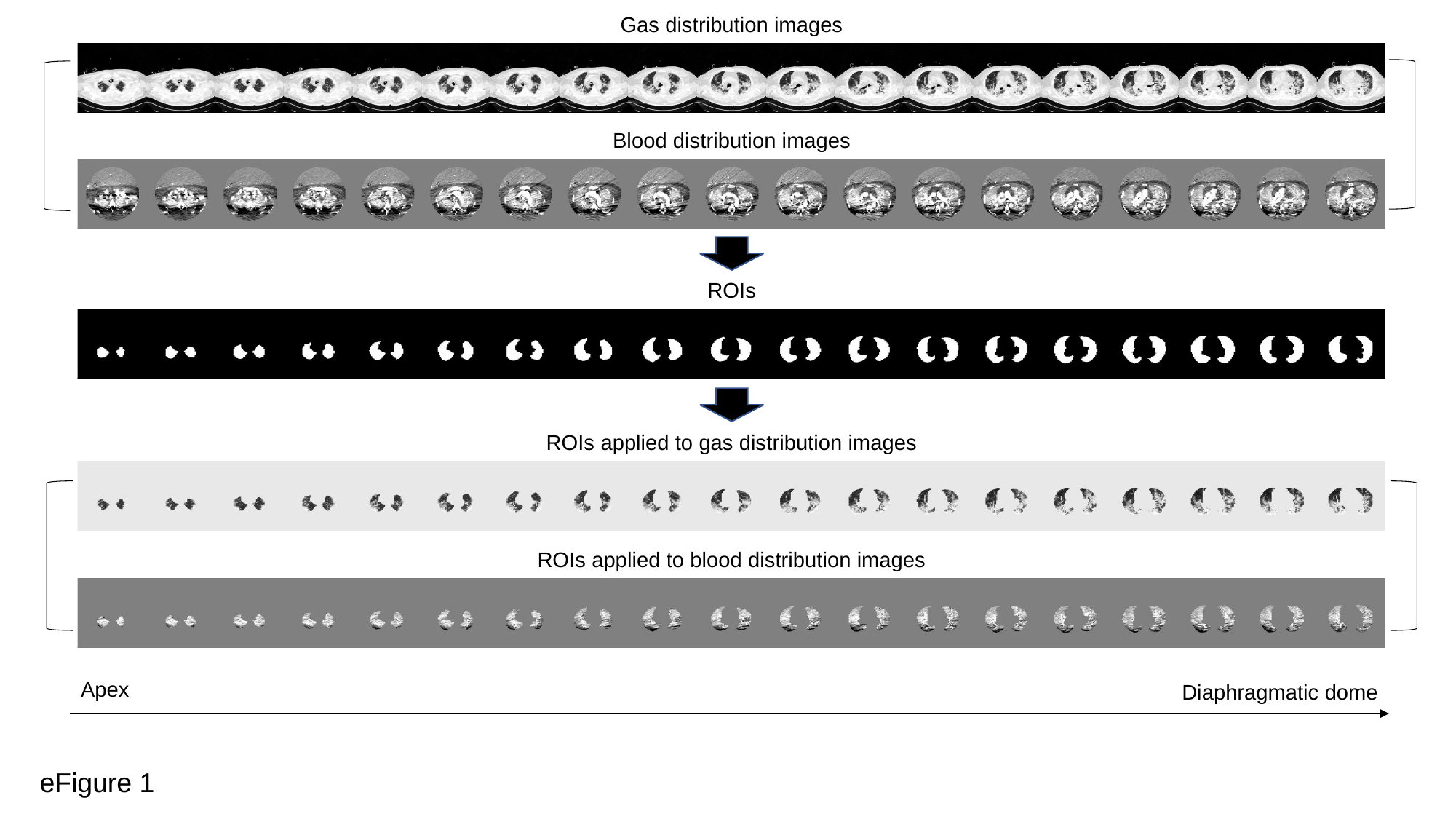

Gas distribution images
Blood distribution images
ROIs
ROIs applied to gas distribution images
ROIs applied to blood distribution images
Apex
Diaphragmatic dome
eFigure 1

## Slide 2
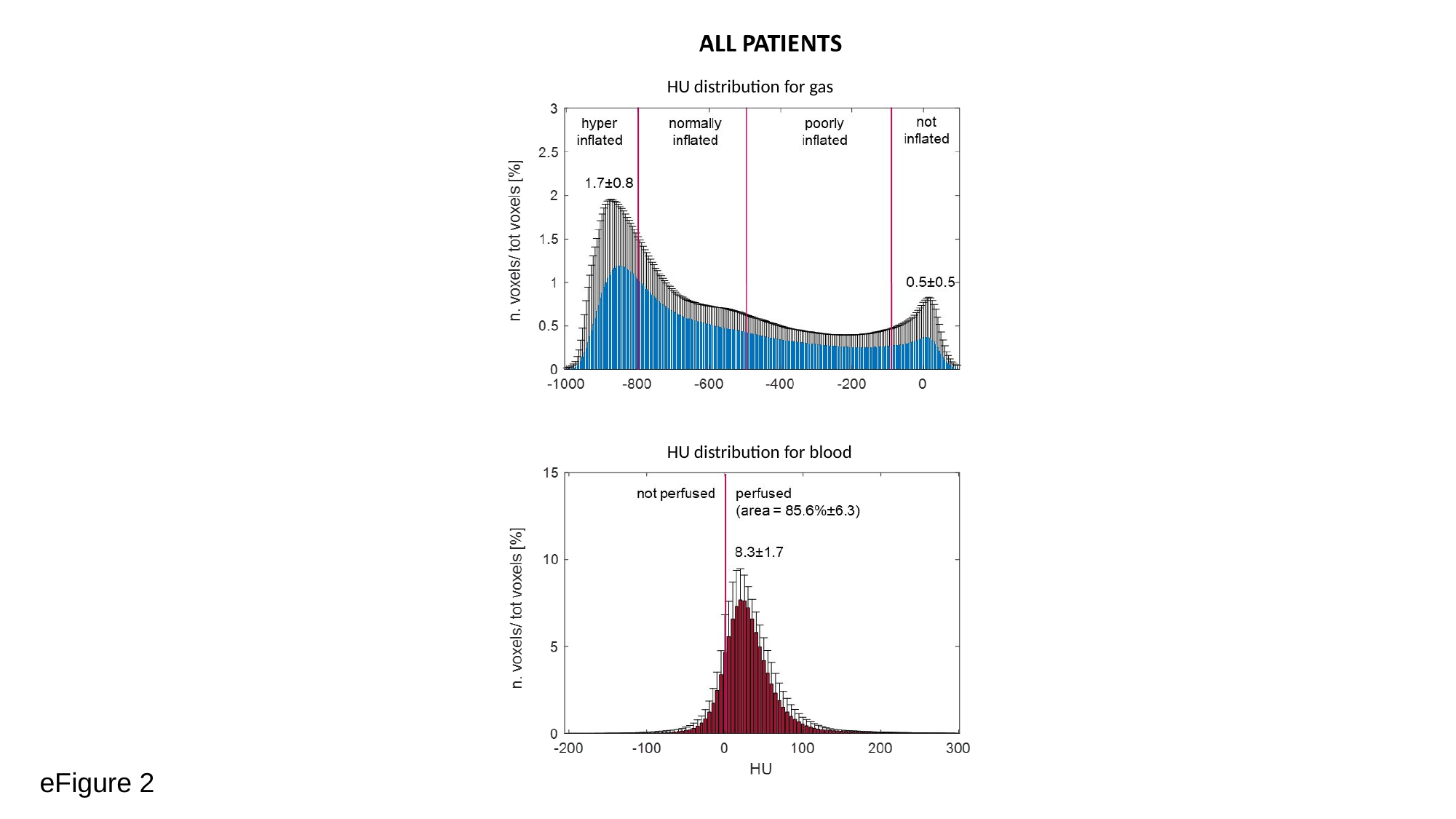

HU distribution for gas
HU distribution for blood
eFigure 2

## Slide 3
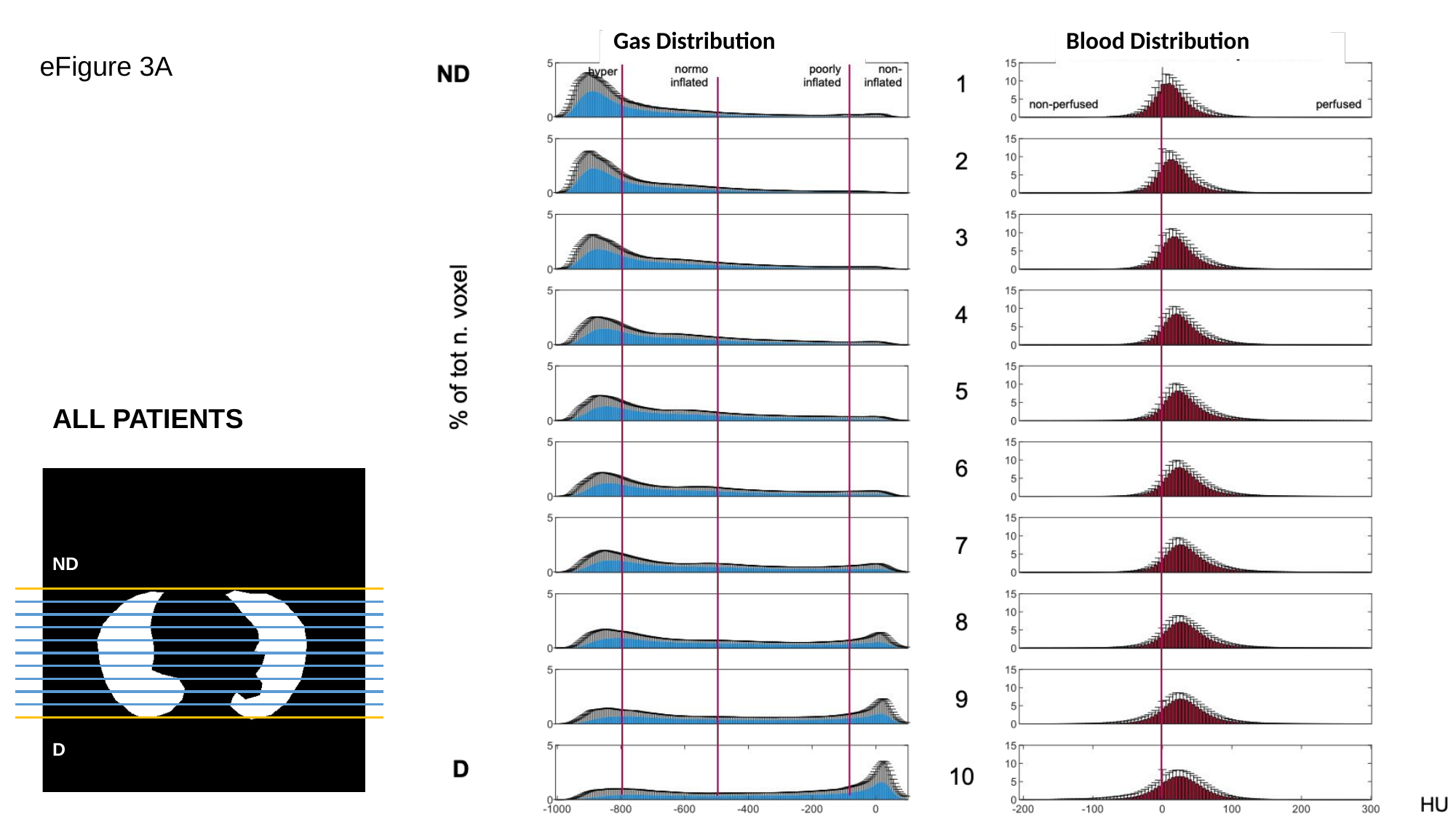

HU distribution for gas
HU distribution for blood
eFigure 3A
ALL PATIENTS
ND
D
Gas Distribution
Blood Distribution

## Slide 4
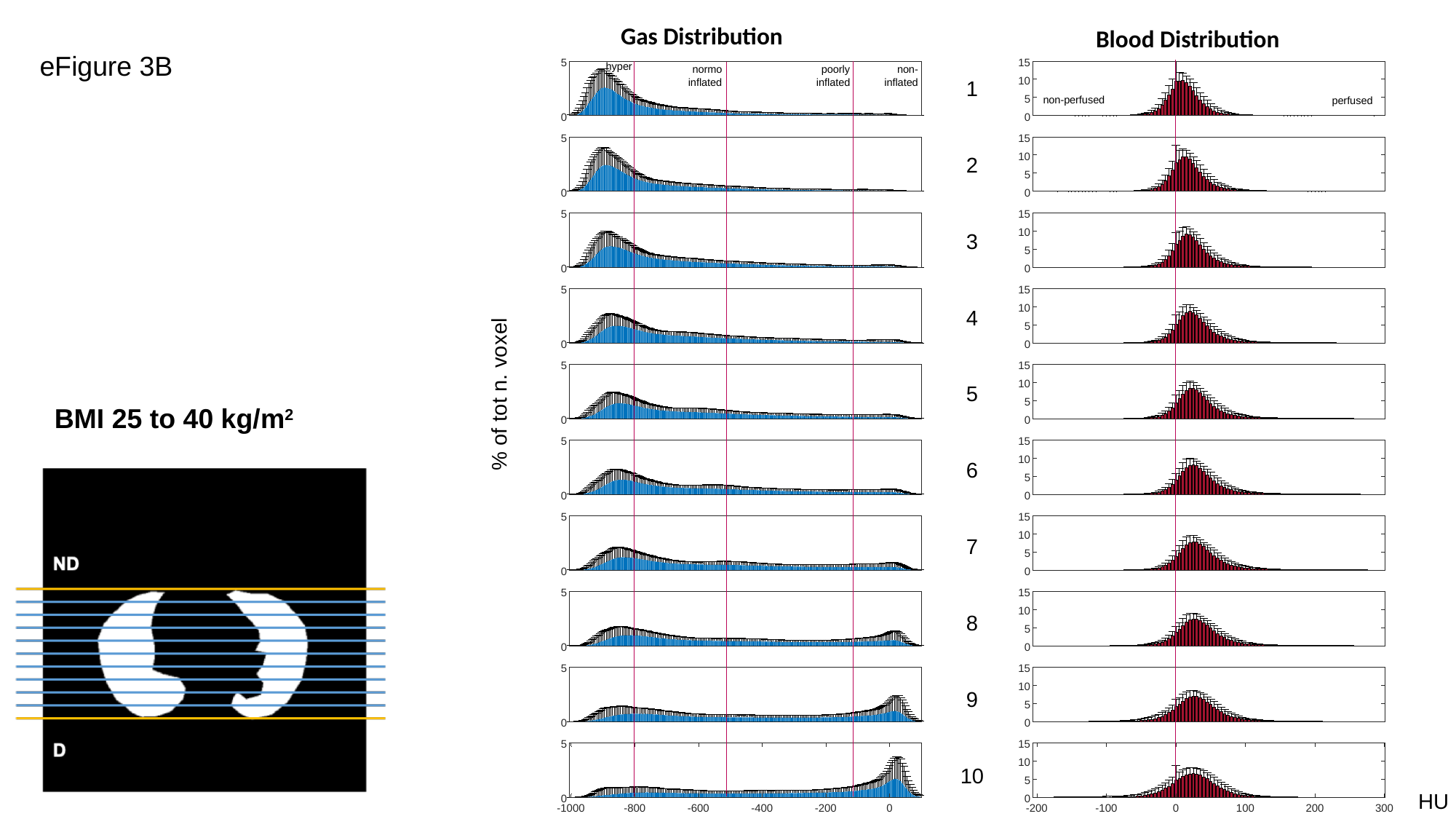

Gas Distribution
Gas Distribution
Blood Distribution
Blood Distribution
Gas Distribution
HU distribution for blood
eFigure 3B
hyper
normo
inflated
poorly
inflated
non-inflated
1
2
3
4
5
6
7
8
9
10
non-perfused
perfused
% of tot n. voxel
BMI 25 to 40 kg/m2
HU

## Slide 5
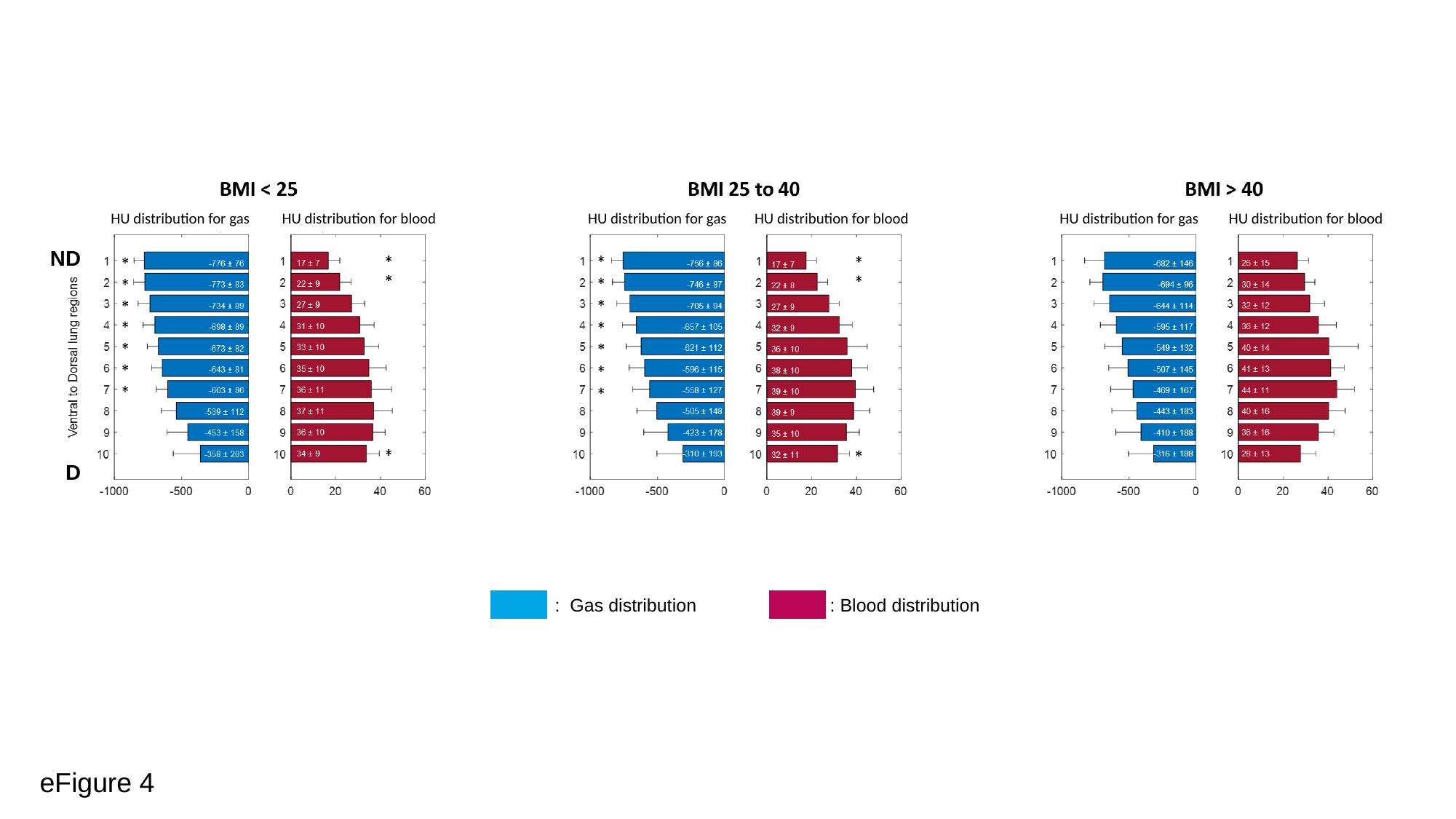

HU distribution for blood
HU distribution for blood
HU distribution for blood
HU distribution for gas
HU distribution for gas
HU distribution for gas
Gas distribution
Blood distribution
Gas distribution
Blood distribution
ND
D
: Gas distribution
: Blood distribution
eFigure 4

## Slide 6
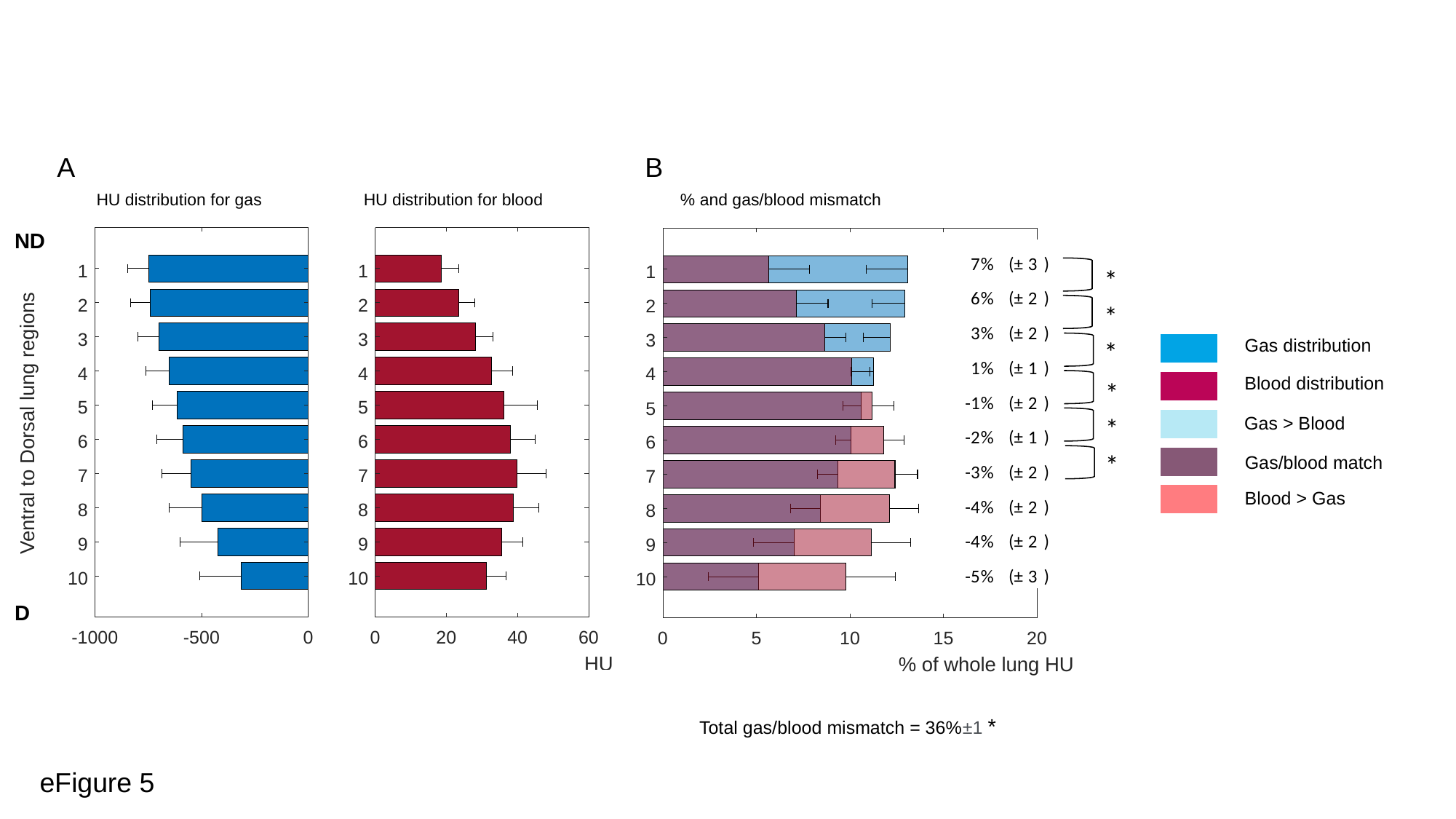

A
B
HU distribution for gas
HU distribution for blood
% and gas/blood mismatch
ND
| 7 | % | (± | 3 | ) |
| --- | --- | --- | --- | --- |
| 6 | % | (± | 2 | ) |
| 3 | % | (± | 2 | ) |
| 1 | % | (± | 1 | ) |
| -1 | % | (± | 2 | ) |
| -2 | % | (± | 1 | ) |
| -3 | % | (± | 2 | ) |
| -4 | % | (± | 2 | ) |
| -4 | % | (± | 2 | ) |
| -5 | % | (± | 3 | ) |
*
*
*
*
*
*
Gas distribution
Blood distribution
Gas > Blood
Gas/blood match
Blood > Gas
D
Total gas/blood mismatch = 36%±1 *
eFigure 5

## Slide 7
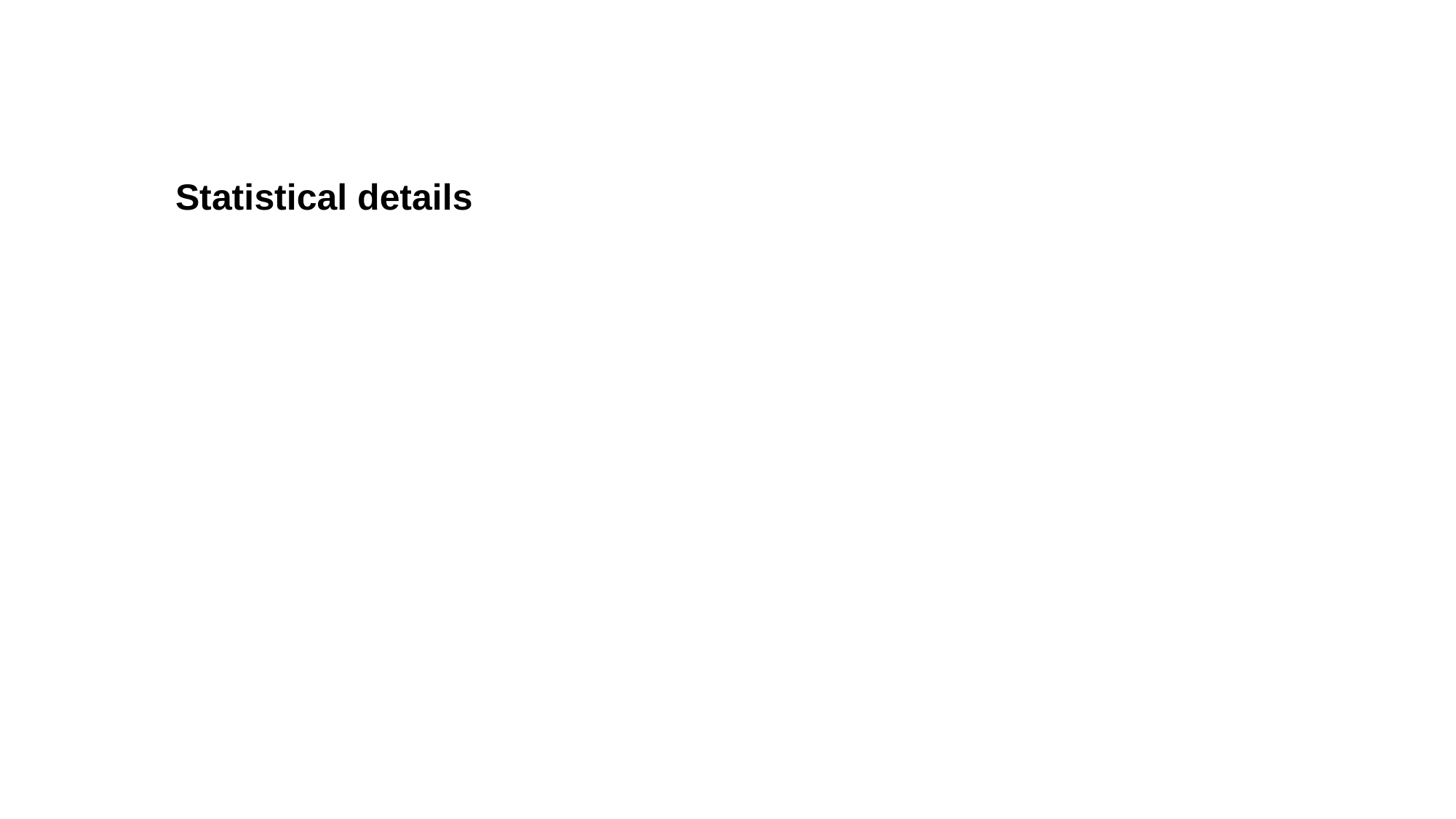

Statistical details

## Slide 8
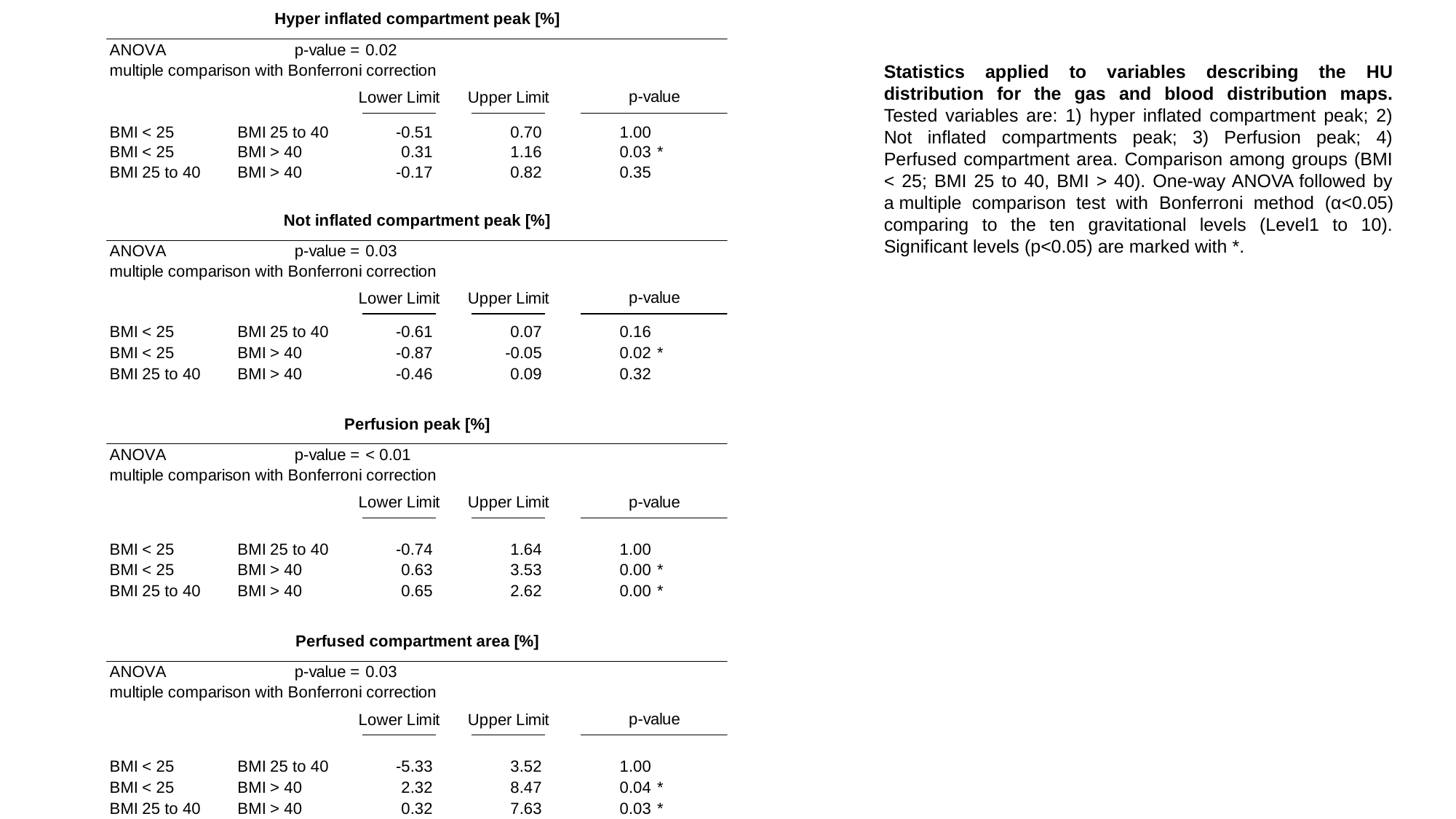

Statistics applied to variables describing the HU distribution for the gas and blood distribution maps. Tested variables are: 1) hyper inflated compartment peak; 2) Not inflated compartments peak; 3) Perfusion peak; 4) Perfused compartment area. Comparison among groups (BMI < 25; BMI 25 to 40, BMI > 40). One-way ANOVA followed by a multiple comparison test with Bonferroni method (α<0.05) comparing to the ten gravitational levels (Level1 to 10). Significant levels (p<0.05) are marked with *.

## Slide 9
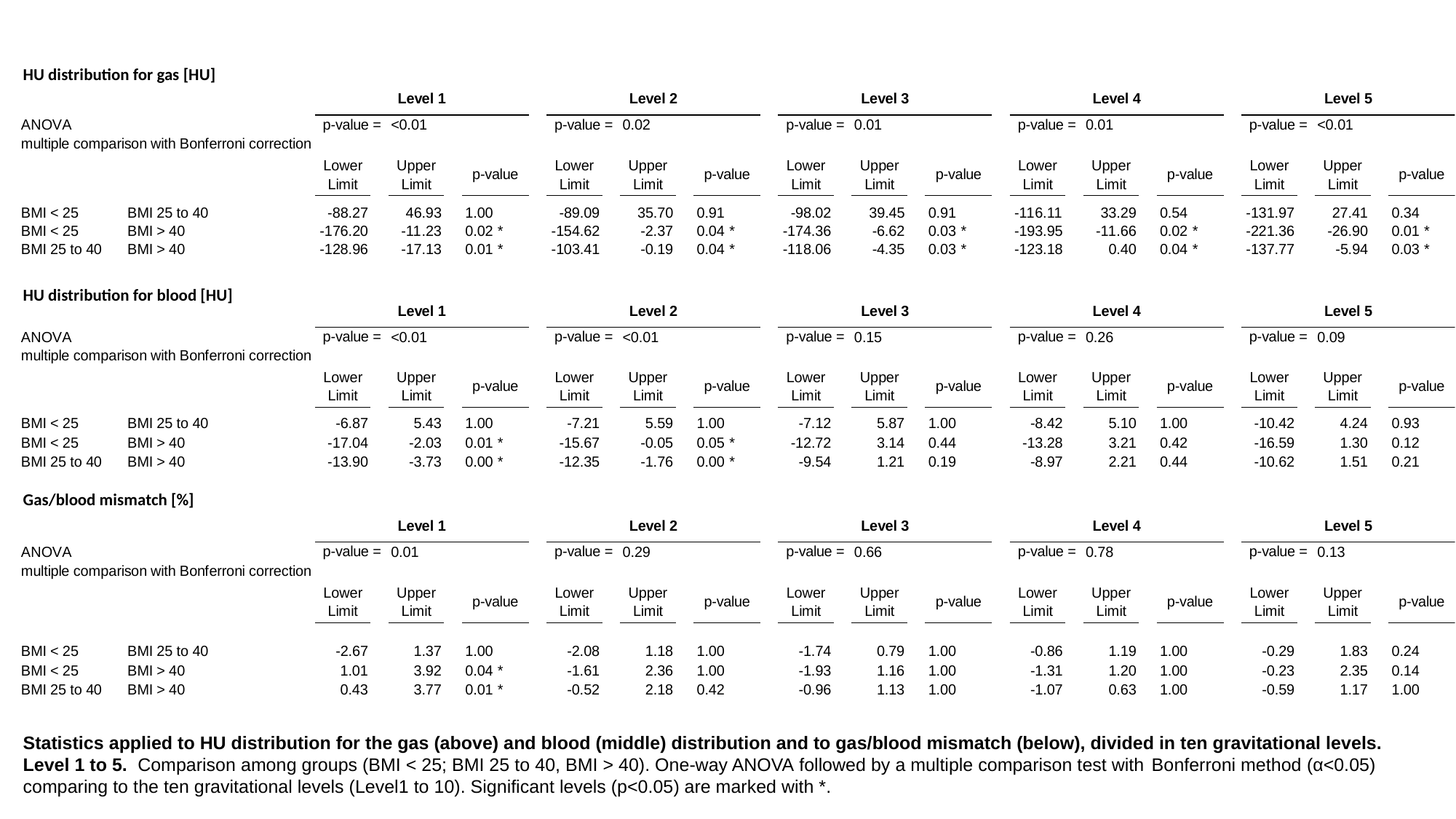

HU distribution for gas [HU]
HU distribution for blood [HU]
Gas/blood mismatch [%]
Statistics applied to HU distribution for the gas (above) and blood (middle) distribution and to gas/blood mismatch (below), divided in ten gravitational levels. Level 1 to 5. Comparison among groups (BMI < 25; BMI 25 to 40, BMI > 40). One-way ANOVA followed by a multiple comparison test with Bonferroni method (α<0.05) comparing to the ten gravitational levels (Level1 to 10). Significant levels (p<0.05) are marked with *.

## Slide 10
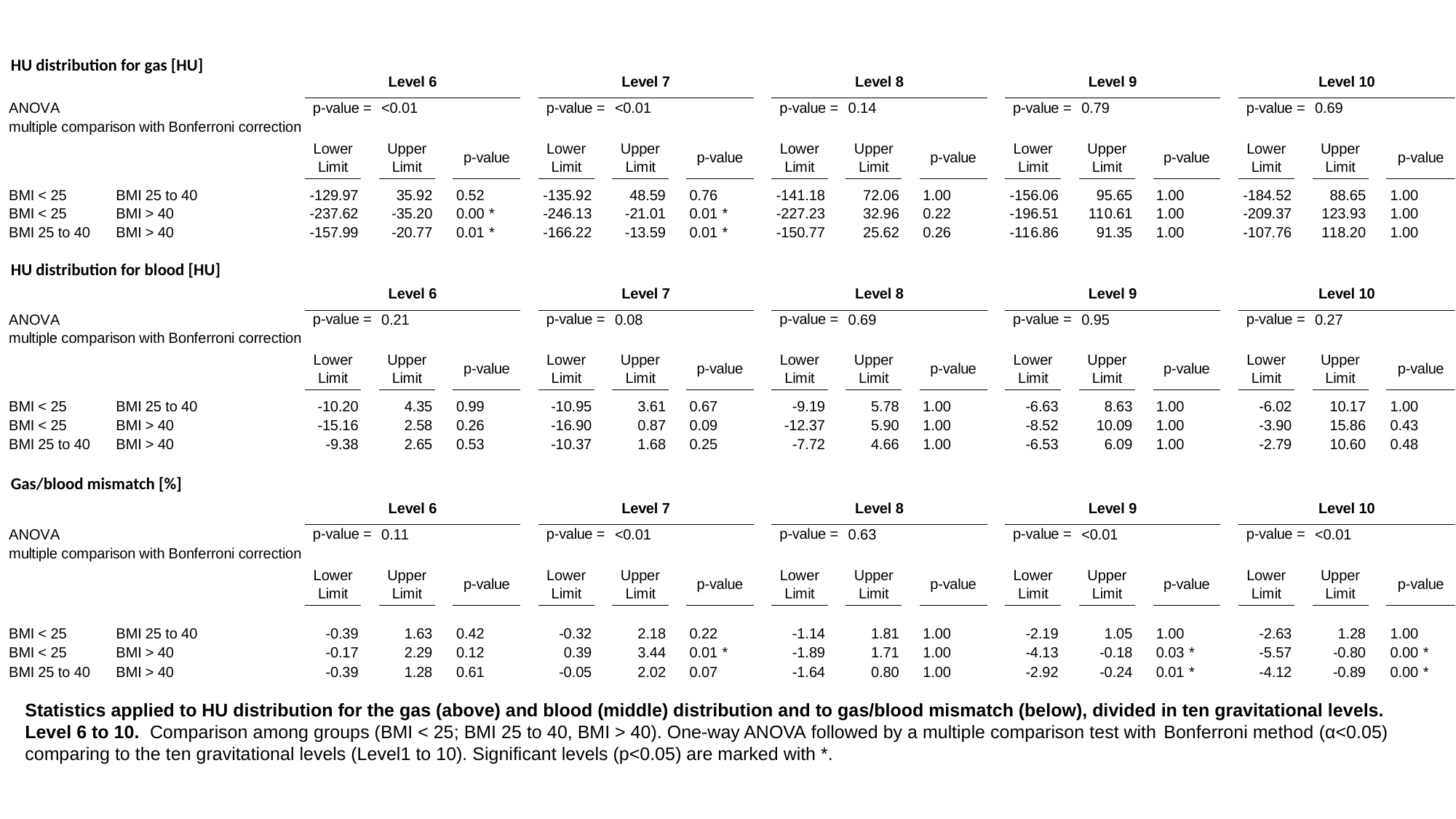

HU distribution for gas [HU]
HU distribution for gas [HU]
HU distribution for blood [HU]
Gas/blood mismatch [%]
Statistics applied to HU distribution for the gas (above) and blood (middle) distribution and to gas/blood mismatch (below), divided in ten gravitational levels. Level 6 to 10. Comparison among groups (BMI < 25; BMI 25 to 40, BMI > 40). One-way ANOVA followed by a multiple comparison test with Bonferroni method (α<0.05) comparing to the ten gravitational levels (Level1 to 10). Significant levels (p<0.05) are marked with *.

## Slide 11
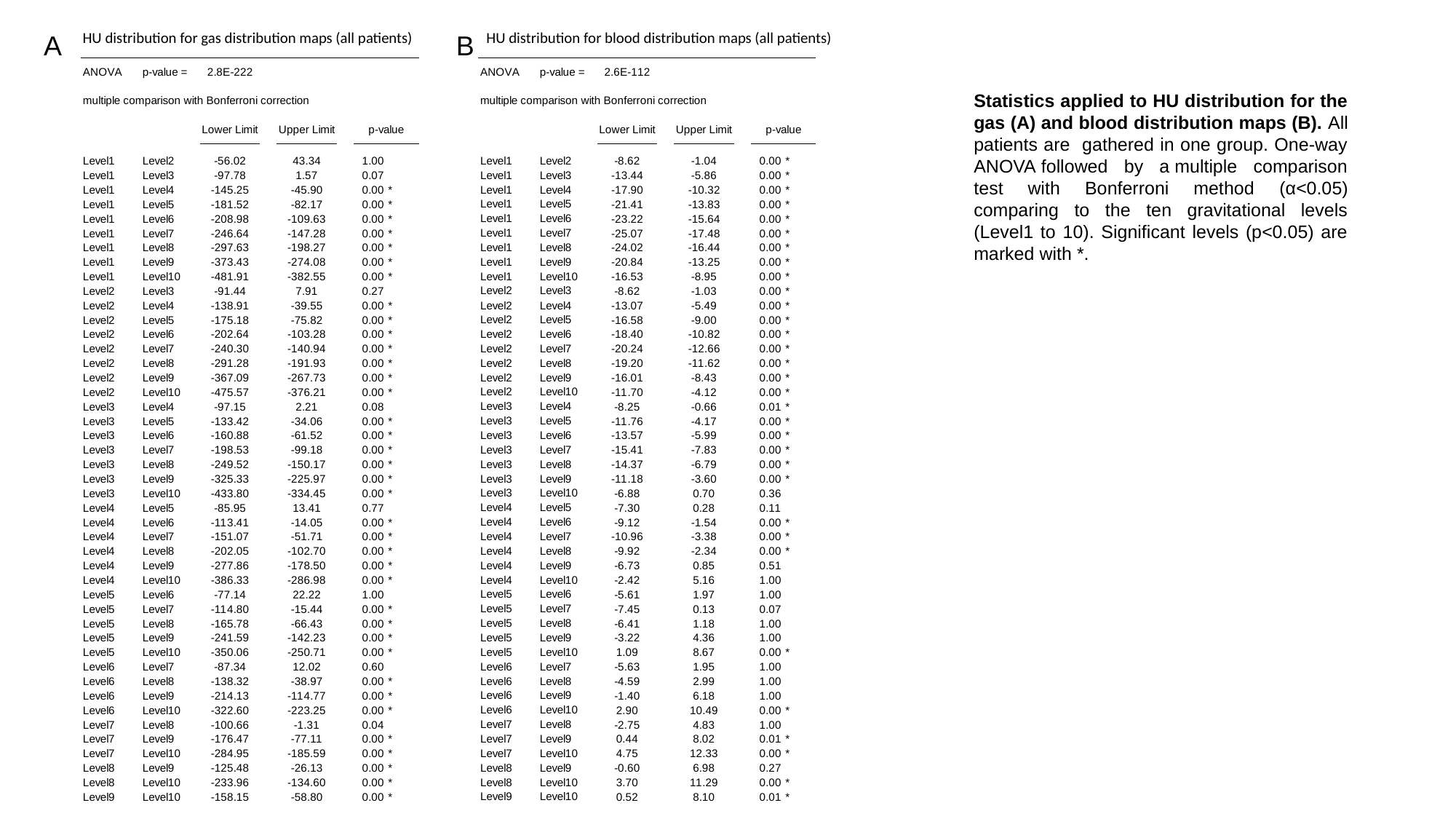

A
HU distribution for gas distribution maps (all patients)
B
HU distribution for blood distribution maps (all patients)
Statistics applied to HU distribution for the gas (A) and blood distribution maps (B). All patients are gathered in one group. One-way ANOVA followed by a multiple comparison test with Bonferroni method (α<0.05) comparing to the ten gravitational levels (Level1 to 10). Significant levels (p<0.05) are marked with *.

## Slide 12
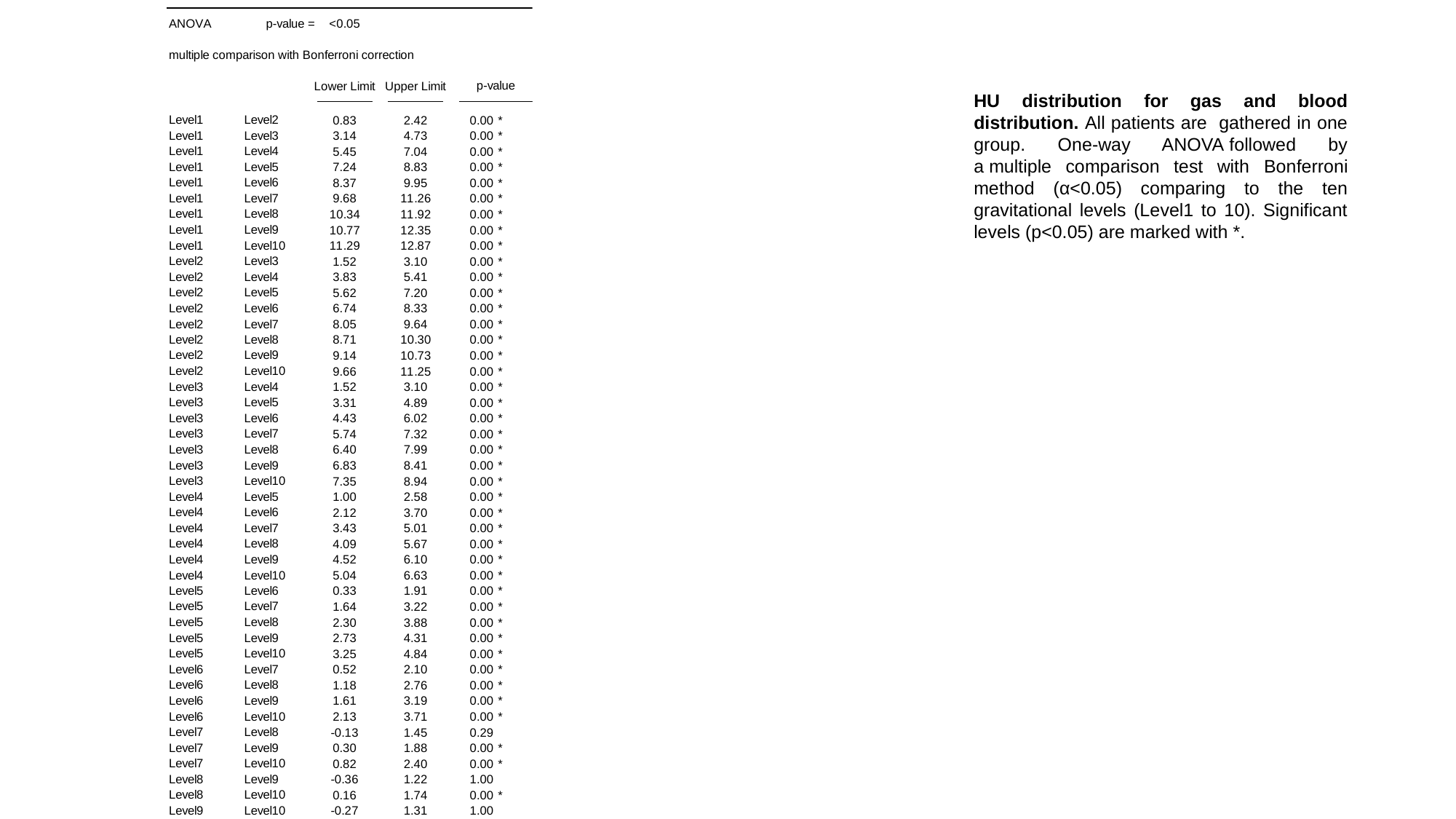

HU distribution for gas and blood distribution. All patients are gathered in one group. One-way ANOVA followed by a multiple comparison test with Bonferroni method (α<0.05) comparing to the ten gravitational levels (Level1 to 10). Significant levels (p<0.05) are marked with *.

## Slide 13
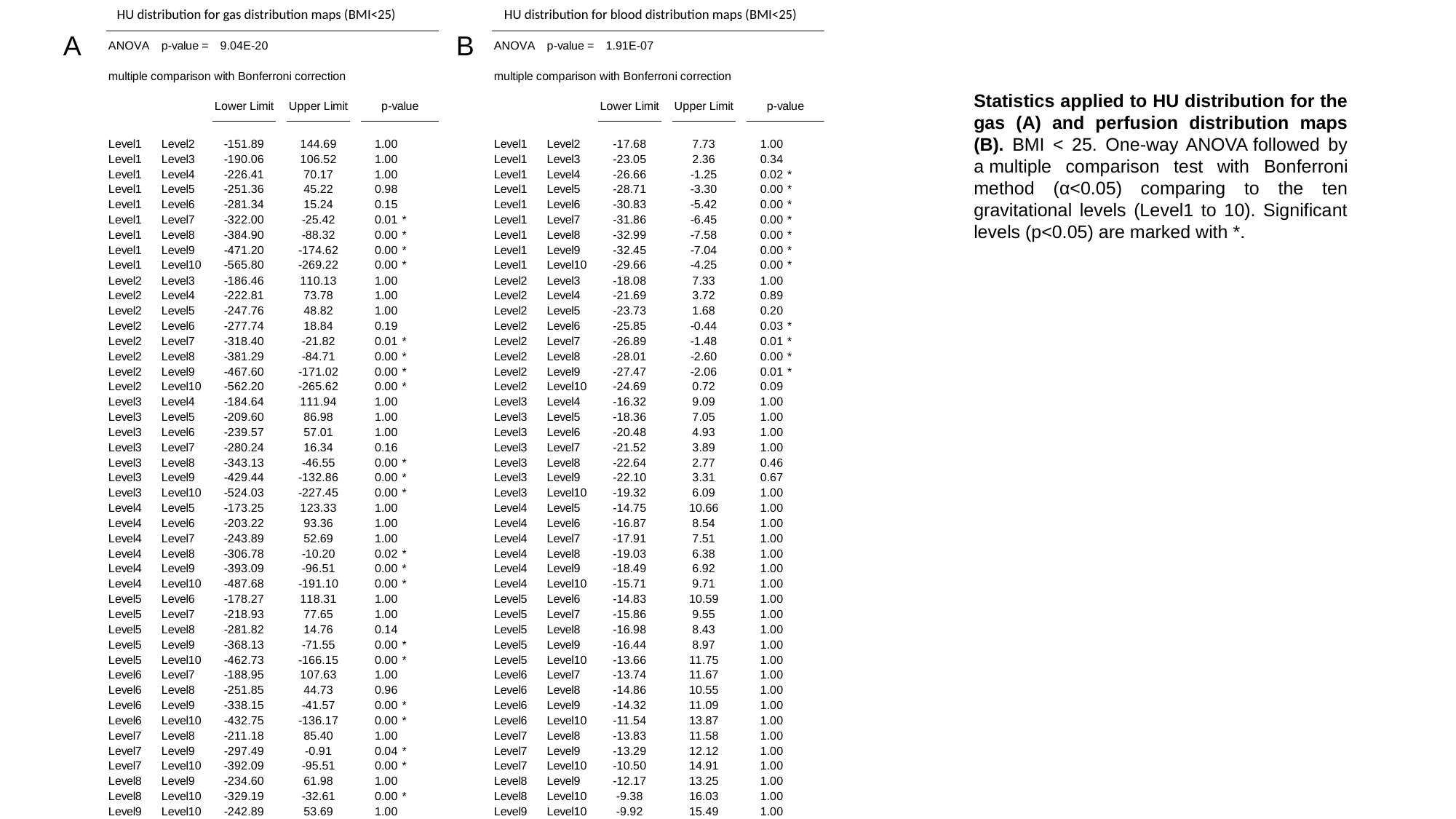

HU distribution for gas distribution maps (BMI<25)
HU distribution for blood distribution maps (BMI<25)
A
B
Statistics applied to HU distribution for the gas (A) and perfusion distribution maps (B). BMI < 25. One-way ANOVA followed by a multiple comparison test with Bonferroni method (α<0.05) comparing to the ten gravitational levels (Level1 to 10). Significant levels (p<0.05) are marked with *.

## Slide 14
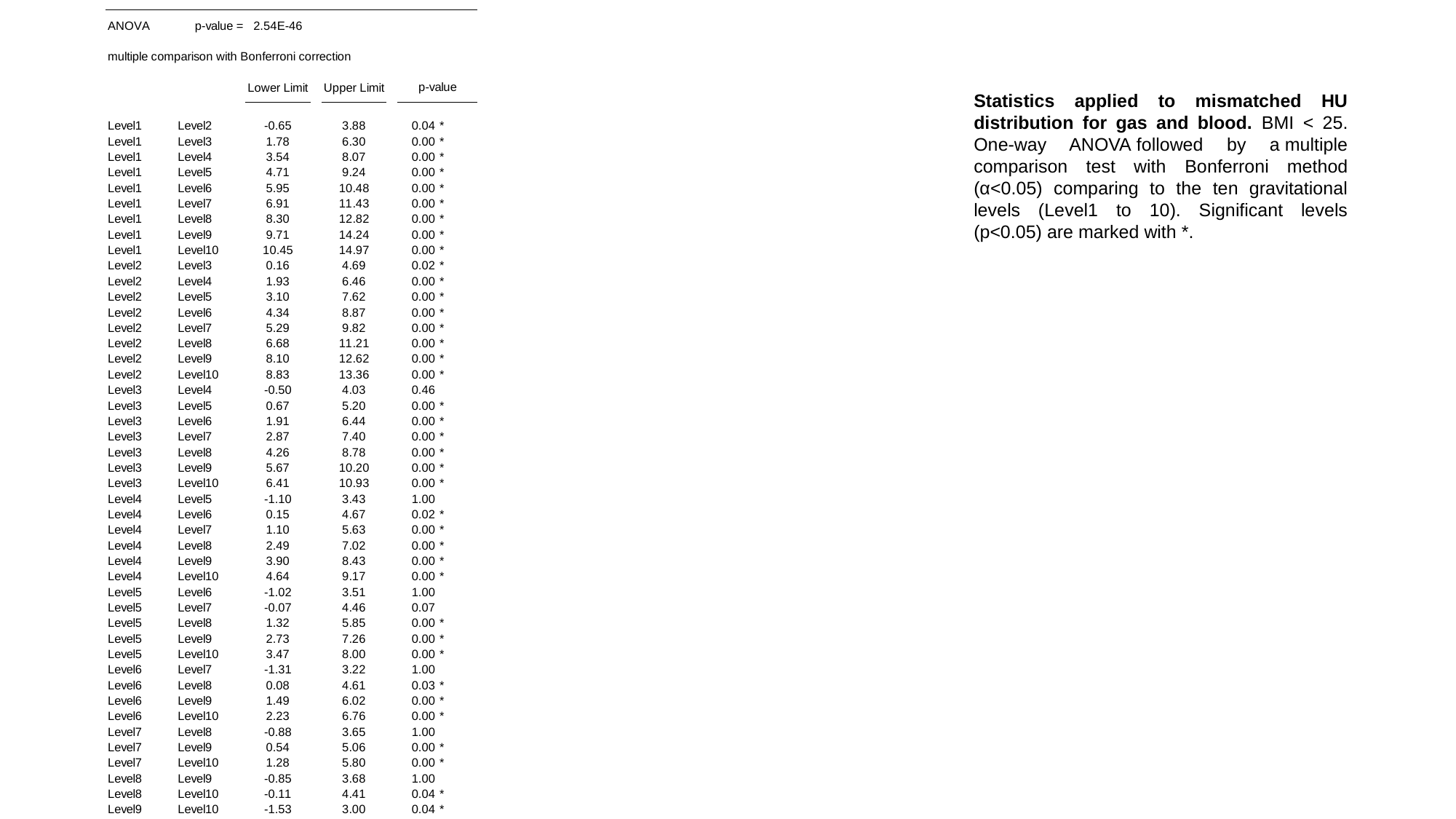

Statistics applied to mismatched HU distribution for gas and blood. BMI < 25. One-way ANOVA followed by a multiple comparison test with Bonferroni method (α<0.05) comparing to the ten gravitational levels (Level1 to 10). Significant levels (p<0.05) are marked with *.

## Slide 15
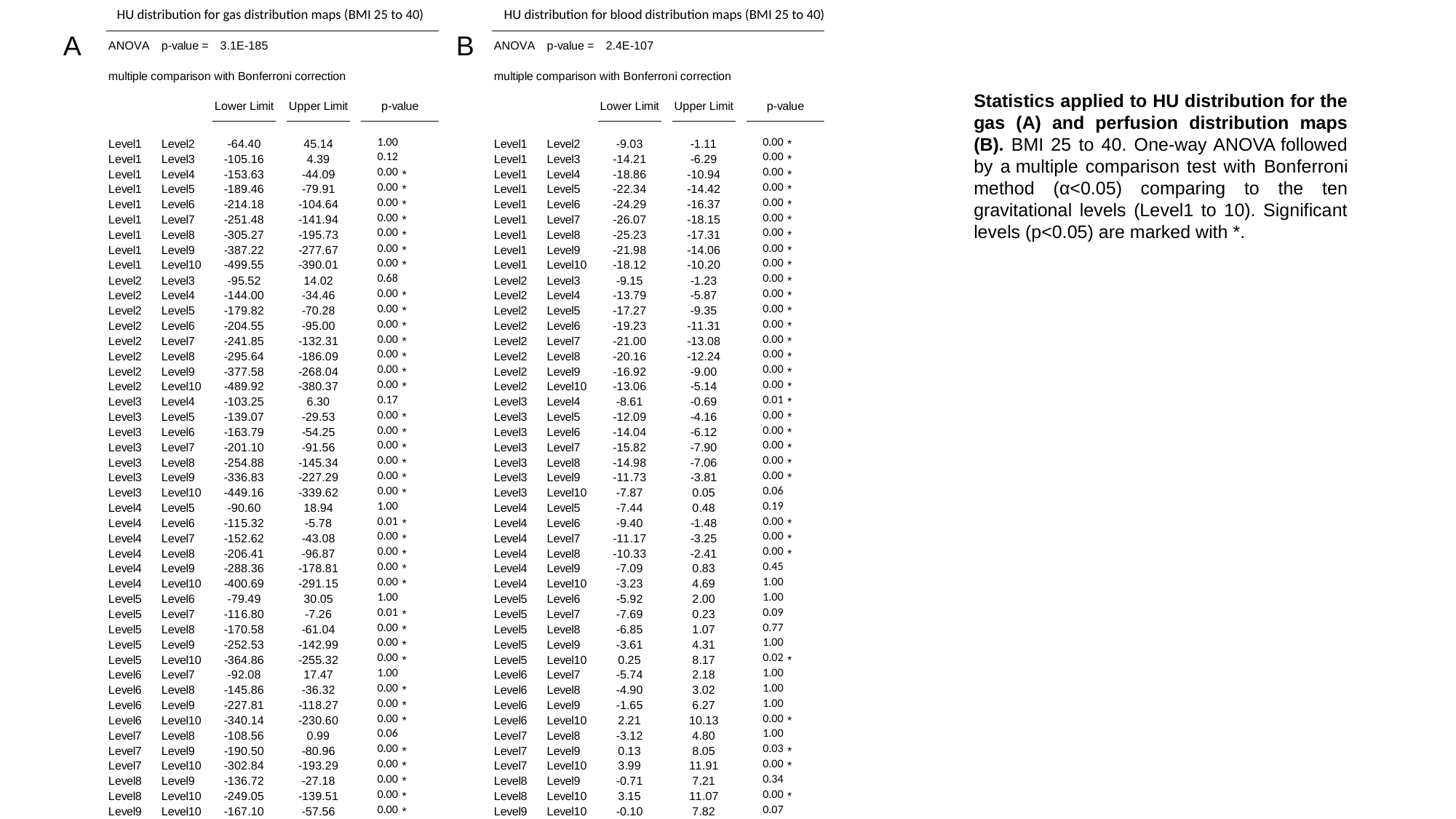

HU distribution for gas distribution maps (BMI 25 to 40)
HU distribution for blood distribution maps (BMI 25 to 40)
A
B
Statistics applied to HU distribution for the gas (A) and perfusion distribution maps (B). BMI 25 to 40. One-way ANOVA followed by a multiple comparison test with Bonferroni method (α<0.05) comparing to the ten gravitational levels (Level1 to 10). Significant levels (p<0.05) are marked with *.

## Slide 16
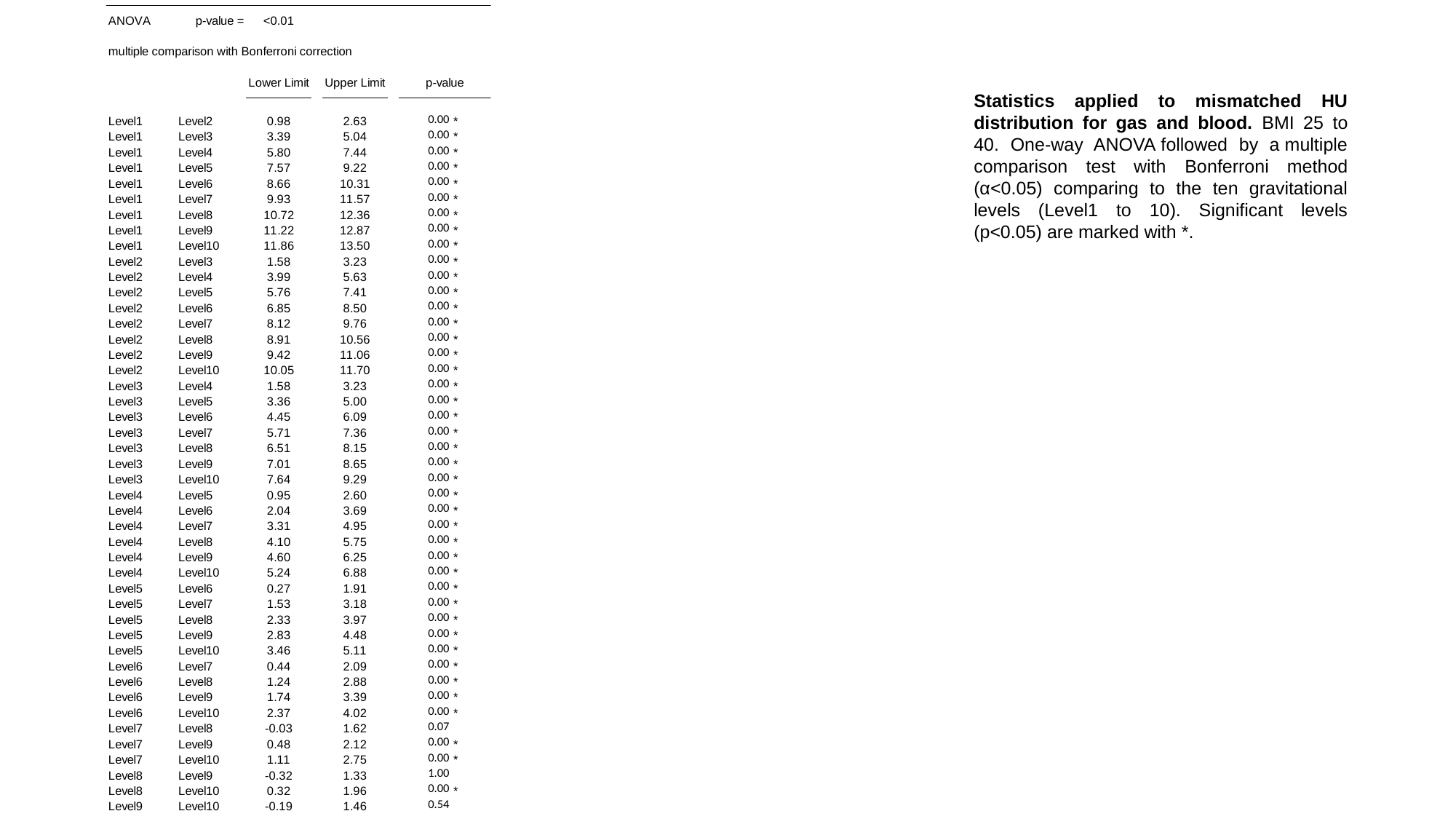

Statistics applied to mismatched HU distribution for gas and blood. BMI 25 to 40. One-way ANOVA followed by a multiple comparison test with Bonferroni method (α<0.05) comparing to the ten gravitational levels (Level1 to 10). Significant levels (p<0.05) are marked with *.

## Slide 17
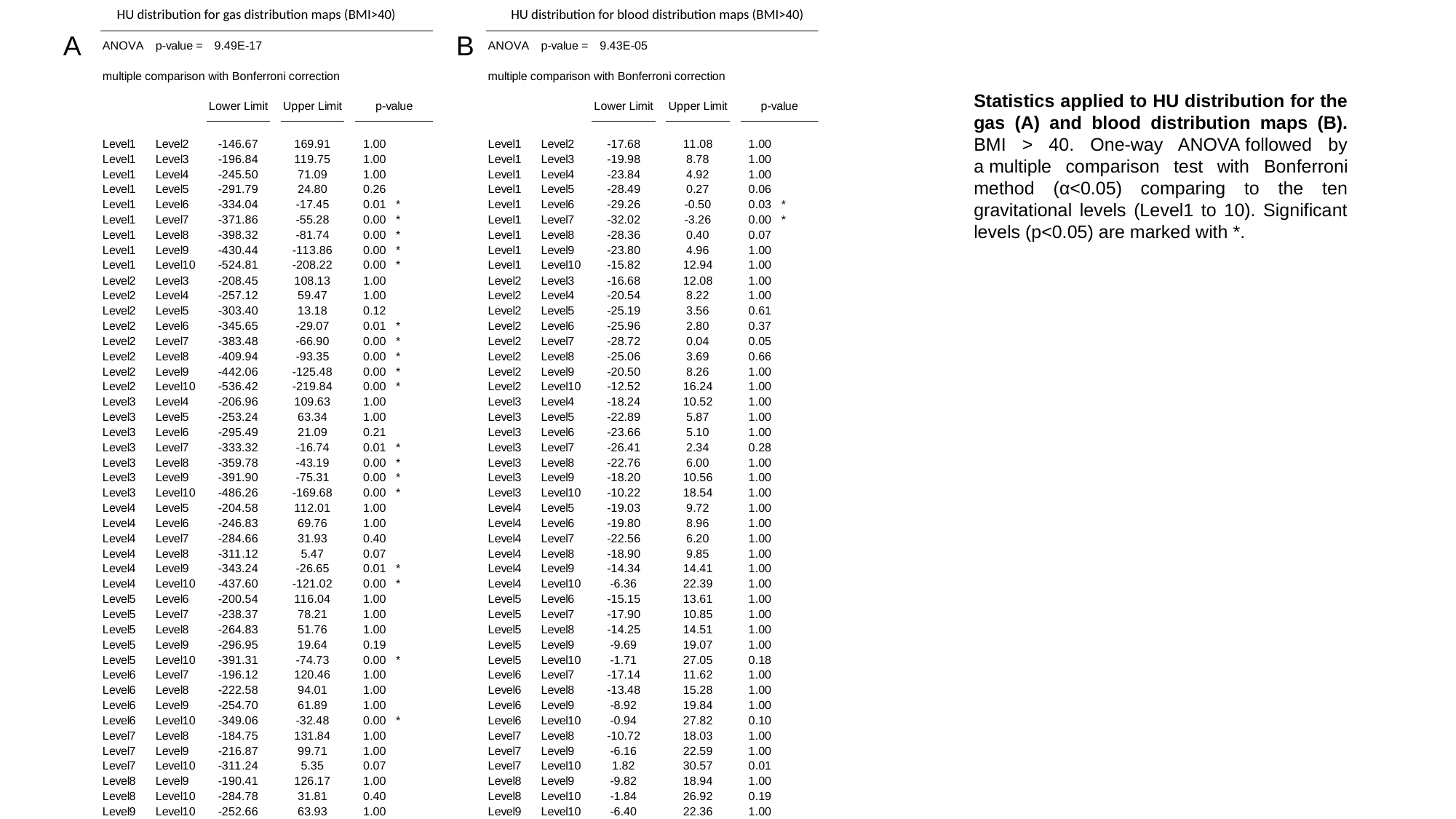

HU distribution for gas distribution maps (BMI>40)
HU distribution for blood distribution maps (BMI>40)
A
B
Statistics applied to HU distribution for the gas (A) and blood distribution maps (B). BMI > 40. One-way ANOVA followed by a multiple comparison test with Bonferroni method (α<0.05) comparing to the ten gravitational levels (Level1 to 10). Significant levels (p<0.05) are marked with *.

## Slide 18
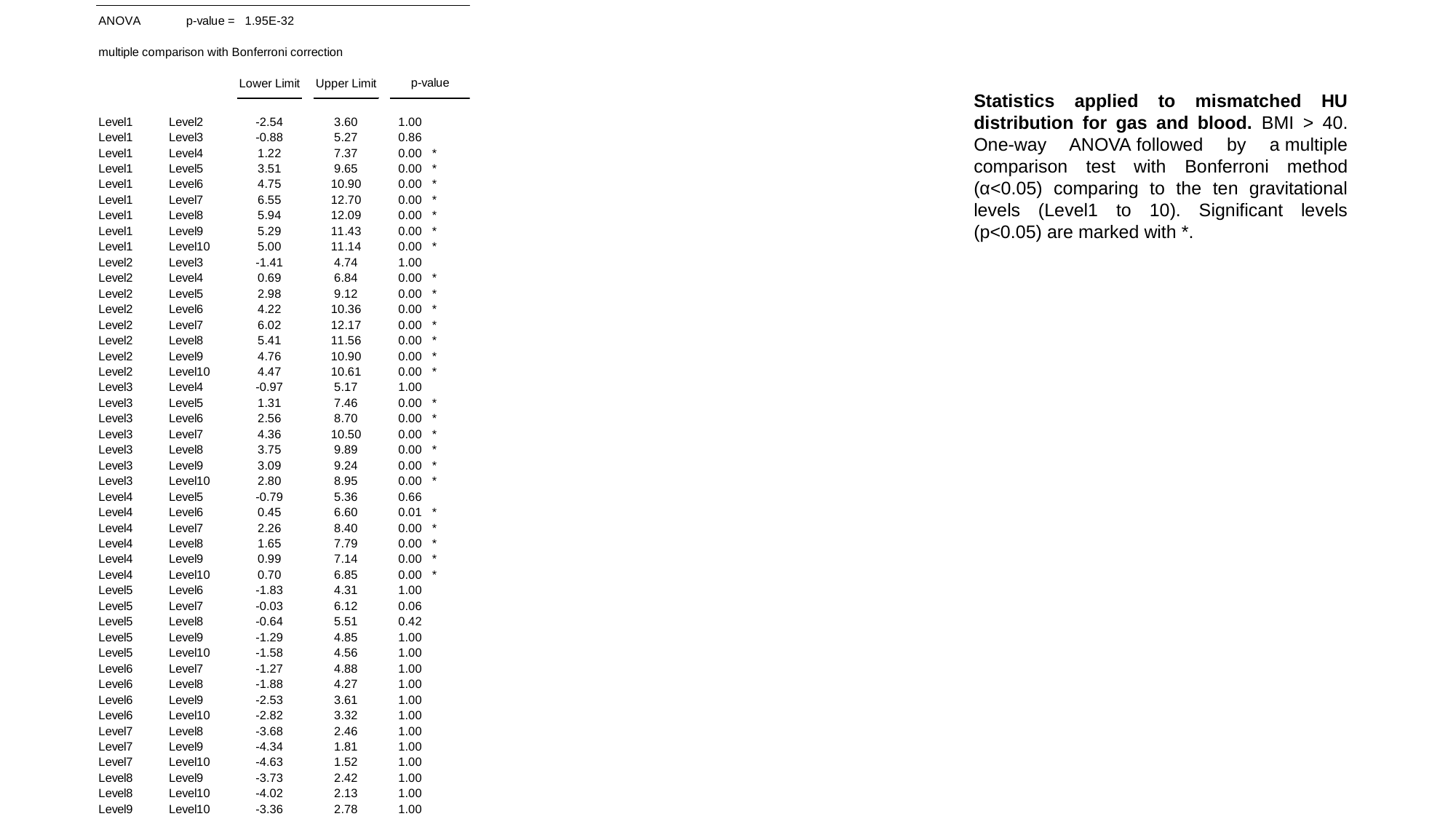

Statistics applied to mismatched HU distribution for gas and blood. BMI > 40. One-way ANOVA followed by a multiple comparison test with Bonferroni method (α<0.05) comparing to the ten gravitational levels (Level1 to 10). Significant levels (p<0.05) are marked with *.
