# Supplementary material for: Body mass index is associated with pulmonary gas and blood distribution mismatch in COVID-19 acute respiratory failure. A physiological study
Source: Front Physiol. 2024 Jul 10;15:1399407. doi: 10.3389/fphys.2024.1399407 (PMC11266150; doi:10.3389/fphys.2024.1399407)
Supplement: Supplementary file 2 [file DataSheet1.pdf]

Body mass index is associated with pulmonary gas and blood distribution mismatch in COVID-19 acute respiratory failure. A physiological study

Kristin J Bjarnadottir[1*]; Gaetano Perchiazzi[1,2*]; Caroline Lördal Sidenbladh[3]; Aleksandra Larina[1]; Ewa Wallin[1]; Ing-Marie Larsson[1]; Stephanie Franzén[1]; Anders O Larsson[9]; Mayson LA Sousa[4,5,6]; Monica Segelsjö[7]; Tomas Hansen[7]; Robert Frithiof[1]; Michael Hultström[1,8]; Miklos Lipcsey[1,2]; Mariangela Pellegrini[1,2].

*Shared first authorship

Online data supplement

METHODS

Study design

This is a single-centre prospective observational study. The National Ethical Review Agency approved the study Dnr 2017-043 (with amendments 2019-00169, 2020-01623, 2020-02719, 2020-05730, 2021-01469) as well as de-novo application Dnr 2022-00526-01 pertaining to the acute study and Dnr 2020-02697 (with amendments 2020-03629, 2020-05758, 2021-02205, 2022-01115-02) pertaining to the follow-up study. Written informed consent was obtained from the patients when possible. Otherwise, informed consent was initially asked from next of kin and later confirmed by patients, if feasible. The study was registered a priori (clinicaltrials.gov: NCT04316884, registered March 13, 2020, and NCT04474249, registered July 16, 2020). General inclusion criteria for the prospective study were 1) age >18 years; 2) a positive polymerase chain reaction test for SARS-CoV2 on a nasal swab specimen; 3) ARF as the main cause of ICU admission, combined with the need for any kind of respiratory support;

4) admission to an ICU at Uppsala University Hospital (Sweden); and 5) at least one chest DECT performed on clinical indication during the ICU stay. For the present analysis, at least one chest dual-energy computed tomography (DECT) performed during ICU stay was required for patient enrollment. Body mass index (BMI) was considered both as a continuous and as a categorical variable and divided into three subgroups defined based on patient BMI at ICU admission: 1) BMI<25 kg/m^2 (non-obese); 2) BMI 25–40 kg/m^2 (overweight and obese); or 3) BMI>40 kg/m^2 (morbidly obese) (*Figure 1)*. STROBE guidelines were followed for data reporting. Power calculation was not conducted before the study, and the sample size was based on the available data.

Clinical data

Comprehensive clinical data were collected on ICU admission, at ICU discharge and on the day of DECT scanning.

DECT scans

DECT imaging was performed on a Siemens Definition Flash dual-source scanner (Siemens Healthineers, Erlangen, Germany). The DECT scans were performed on patients in a supine position with non-ionic iodinated contrast iohexol (Omnipaque 350 mg/ml, GE Healthcare, USA) injected in a peripheral vein at a rate of 4 ml/s with a power injector (Stellant D, Medrad Inc, Indianola, PA, USA) with a total volume of 60–80 ml, according to patient size, with a 50 ml saline flush. To estimate optimal scan delay, test-bolus was performed with a small amount of contrast media (10 ml, injected at 4 ml/s). A region of interest in the pulmonary trunk generated a time-enhancement curve using DynEVA (scanner software). Scanstart was defined as peak +7 s. The scans were acquired in static breath hold, covering the whole lung

parenchyma. To avoid streak artifacts due to highly concentrated contrast media in the superior vena cava territory, scans were acquired in the caudocranial direction. The dual-energy protocol setup was 80/Sn 140 kV automatic tube current modulation (Care Dose 4D) with a quality reference of 190 mAs on tube A (automatically given 81 mAs on tube B), collimation 64×0.6 mm, rotation time 0.33 s, and pitch 0.55. Increased tube voltage 100/140Sn was needed for larger patients (above ~90 kg), automatically given a reference of 96/81 mAs. The mean CTDIvol was 15.7±7.0 mGy (*Figure 2*). The quality of each collected DECT scan was confirmed by one of the authors (TH), a radiologist specialized in thoracic radiology. As the beam rotates around a subject, it exposes a block of tissue from multiple directions. Each voxel is given a density value by using a mathematical process called Fourier analysis at its given position, expressed as a CT number. The CT number is determined by the tissue's attenuation coefficient (μ) which, in turn, depends on the density and atomic number of the material and the energies of the X-ray photon used. Changing the X-ray photon energy will result in a different attenuation coefficient for the same tissue, which is the concept that underlies the DECT technique. All the collected DECT scans were acquired based on clinical indications.

DECT data analysis

The images obtained were stored and processed with DECT post-processing software (Syngo.via, version VB50, Siemens Healthcare), and lung analysis was applied to visualize and quantify the iodine uptake in the lung parenchyma. Two reconstructions from each DECT scan were obtained: 1) virtual non-contrast (VNC) images and 2) virtual contrast (VC) images, mix 0.8 (reconstruction consisting of 80% from tube A with low kV to highlight the iodine contrast), corresponding to the gas and blood distribution maps, respectively. The images were reconstructed into two-dimensional square matrices of 512 × 512 and three-dimensional voxels with dimensions of 0.7461 mm × 0.7461 mm × 1 mm. For each DECT examination, 19 pairs

of images, each pair counting gas and blood distribution, were selected along the cranial–caudal axis and uniformly spaced between the apex of the lung and the diaphragmatic dome (*Figure 2* and *eFigure 1*). All the selected images underwent a semiautomatic delineation of the regions of interest corresponding to the lung parenchyma (*Figure 2*). The resulting gas and blood distribution maps were subsequently divided into ten gravitational levels, as illustrated in *Figure 2*. Each image voxel attained a representative CT number in Hounsfield units (HU), which relate to the tissue's ability to attenuate X-rays, where water has 0 HU, air has −1000 HU, and the lung parenchyma, characterized by different degrees of regional aeration, is described by a HU range of between −1000 and 200 HU. Pulmonary gas distribution, estimated from the VNC images, showed a HU range of between −1000 and 100 HU. Pulmonary blood distribution estimated from the VC images showed a HU range of between −200 and 200 HU. Based on previous literature, the HU distribution of the gas distribution maps was classified into four lung compartments(Gattinoni et al., 2001), namely hyperinflated (−1000 to −800 HU), normoinflated (−800 to −500 HU), poorly inflated (−500 to −100 HU), and noninflated (−100 to +100 HU). The HU distribution for the blood distribution maps was instead classified into two compartments: not-perfused (≤0 HU) and perfused (>0 HU) lung.(Uhrig et al., 2015; Ball et al., 2021; Perchiazzi et al., 2022) The HU distribution was analysed based on a histogram (bin breadth of 5 HU) and expressed as an absolute value (number of voxels) as well as a percentage of the total amount of voxels. For each HU distribution profile, the following parameters were collected: 1) the HU peak of the hyperinflated lung; 2) the HU peak of the noninflated lung; 3) the HU peak of perfusion; and 4) the area under the curve for the perfused compartment. For both the pulmonary gas and blood distribution images, the global and regional HU distributions, as well as their mean values for ten gravitational levels, were calculated (*Figure 4-5, and eFigure 2*). The mean HU value for each of the ten gravitational levels of the lung parenchyma allowed a regional analysis of HU distribution (*eFigures 4-5*).

Each regional HU mean was then expressed as a percentage of the whole lung HU (*Figure 6, and eFigures 5*). Based on this, and on the assumption that 1% of regional HU for gas distribution maps correspond to 1% of regional HU for blood distribution maps, an estimation of regional gas/blood mismatch was possible. A linear regression model was applied to BMI and gas/blood mismatch (mismatch = a * BMI + b; where a was the estimated slope, and b the estimated intercept on the y-axis), and used to describe the relation between BMI and gas/blood mismatch as continuous variables.

The whole analysis was conducted using MatLab (Image Processing, Statistics and Machine Learning Toolbox, Release 2022b, The MathWorks, Natick, USA).

REFERENCES

Ball, L., Robba, C., Herrmann, J., Gerard, S. E., Xin, Y., Mandelli, M., et al. (2021). Lung distribution of gas and blood volume in critically ill COVID-19 patients: a quantitative dual-energy computed tomography study. *Crit. Care Lond. Engl.* 25, 214. doi: 10.1186/s13054-021-03610-9

Gattinoni, L., Caironi, P., Pelosi, P., and Goodman, L. R. (2001). What has computed tomography taught us about the acute respiratory distress syndrome? *Am. J. Respir. Crit. Care Med.* 164, 1701–1711. doi: 10.1164/ajrccm.164.9.2103121

Perchiazzi, G., Larina, A., Hansen, T., Frithiof, R., Hultström, M., Lipcsey, M., et al. (2022). Chest dual-energy CT to assess the effects of steroids on lung function in severe COVID-19 patients. *Crit. Care Lond. Engl.* 26, 328. doi: 10.1186/s13054-022-04200-z

Uhrig, M., Simons, D., Ganten, M.-K., Hassel, J. C., and Schlemmer, H.-P. (2015). Histogram analysis of iodine maps from dual energy computed tomography for monitoring targeted therapy of melanoma patients. *Future Oncol. Lond. Engl.* 11, 591–606. doi: 10.2217/fon.14.265

Supplementary figures legends

eFigure 1. Methods. Selection of DECT images for gas and blood distribution analysis. For each DECT examination, 19 pairs of images, each accounting for gas and blood distribution, were selected along the cranial–caudal axis and uniformly spaced between the apex of the lung and the diaphragmatic dome.

eFigure 2. HU distribution for the gas (above) and blood (below) distribution maps. Data illustrating the whole lung parenchyma and all patients pulled together. x-axis: HU values; y-axis: percentage of voxels per bin compared to total voxels. Histogram bin width equal to 5 HU; for each bin, the values are reported as mean ± SD.

eFigure 3 (A and B). Gravitational HU distribution for the gas (left) and blood (right) distribution maps. All patients pooled together (A) and for the BMI 25-40 group (B). Data illustrating the ten gravitational levels. Histogram bin width equal to 5 HU; y-axis: percentage of total voxels [mean ± SD]. Abbreviations: ND: non-dependent; D: dependent; HU: Hounsfield unit.

eFigure 4. Pulmonary gas and blood distribution analysis for the three BMI groups. HU mean [SD] divided into ten gravitational levels for gas (blue) and blood (red) distribution. Compared to groups with BMI<40, the BMI>40 group showed less gas distribution in the non-dependent regions (Levels 1–7) and greater blood distributionin the most extreme regions of the lung (levels 1, 2, and 10).

eFigure 5. Gas/blood mismatch analysis for the whole patient cohort. A. HU mean [SD] divided into ten gravitational levels for gas (blue) and blood (red) distribution. B. Regional

distribution divided into ten gravitational levels for gas/blood mismatch , where gas exceeding blood in blue and blood exceeding gas content in pink. * To mark differences for the mismatched areas ($p>0.05$). ANOVA followed by multiple comparison with Bonferroni correction ($\alpha<0.05$). Abbreviations: ND: non-dependent; D: dependent; HU: Hounsfield unit.
